# Blue organic light-emitting diodes with hybridized local and charge-transfer excited state realizing high external quantum efficiency†

**DOI:** 10.1039/d0ra10934g

**Published:** 2021-02-24

**Authors:** Jayaraman Jayabharathi, Shanmugam Thilagavathy, Venugopal Thanikachalam

**Affiliations:** Department of Chemistry, Annamalai University Annamalai nagar Tamilnadu 608 002 India jtchalam2005@yahoo.co.in

## Abstract

Donor–spacer–acceptor (D–π–A) materials CAPI and CCAPI, with hybridized local and charge transfer (HLCT) emissive states, have been synthesized. The twisting D–π–A architecture promotes the partial separation of HOMO and LUMO, leading to an enhanced % CT component, and the anthracene moiety in CAPI and CCAPI increases the conjugation length, leading to an enhanced % LE component. The non-doped device with CCAPI^b^ shows the blue emission (450 nm) with maximum current efficiency (*η*_c_), power efficiency (*η*_p_), and external quantum efficiency (*η*_ex_) of 16.83 cd A^−1^, 15.32 lm W^−1^, and 12.0%, respectively, as well as exciton utilization efficiency (EUE) of 95% with a luminance of 32 546 cd m^−2^ and a roll-off efficiency of 0.53%. The new design strategy has great potential for developing high-performance blue electroluminescent materials.

## Abstract 2

Donor–spacer–acceptor (D–π–A) materials CAPI and CCAPI, with hybridized local and charge transfer (HLCT) emissive states, have been synthesized.

## Graphical abstract

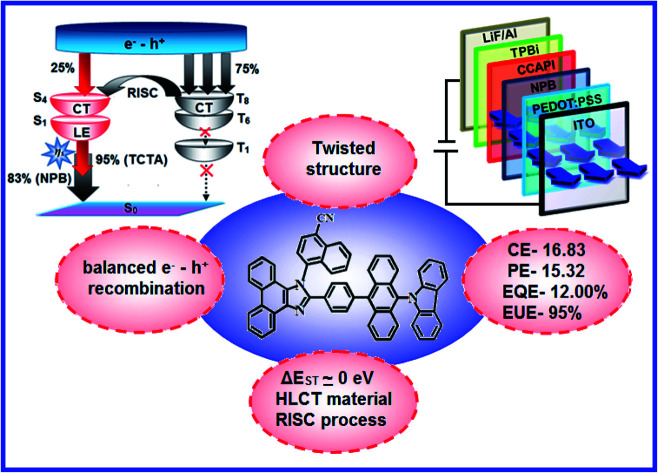

## 1 Introduction

Organic light-emitting diodes (OLEDs) have attracted immense attention in flat-panel displays due to their high efficiency, brightness, wide viewing angle, flexibility and low cost.^1–4^ Efficient pure blue OLEDs have gained both academic and industrial attention due to their short lifetimes, wide bandgaps and low efficiency as compared to their counterparts.^5^ Blue emitters generate high charge injection barriers, leading to unbalanced charge injection and transportation due to a wide bandgap.^6^ Blue PhOLEDs with Ir, Pt and Os complexes exhibit 100% internal quantum efficiency (IQE), however, phosphorescent materials require dispersion in suitable host materials with large bandgaps to reduce the quenching of long-lived triplet excitons.^7^ The phase-separation in host–guest systems diminish device efficiency, and efficient hosts for blue PhOLEDs with high triplet energy also cause complications. Though efficient blue TADF (thermally activated delayed fluorescence) materials have been reported with 100% IQE,^6–8^ only a few of them fulfill the National Television System Committee (NTSC) standard blue CIE of (0.14, 0.08),^9–12^ and TADF materials with intramolecular charge-transfer (ICT) show broad emission results in low colour purity.^13^ Therefore, developing efficient fluorophores is still a great challenge in the field of OLEDs.^14–17^

The non-doped blue devices based on anthracene materials having bulky side chains show high stability and are used as a standard to measure quantum yield (PLQY).^15,17,18^ These materials exhibit good carrier injection and transport properties for balanced carrier recombination.^19,20^ Both phosphorescent and TADF materials show efficiency roll-off due to triplet–triplet annihilation (TTA).^21,22^ Many efficient fluorophores also exhibit efficiency roll-off due to carrier confinement reduction at high current densities with unequal carrier mobility.^23–25^ Therefore, deep blue materials with balanced ambipolar properties are urgently needed to reduce efficiency roll-off. Bipolar imidazole derivatives are not only used as electron transport materials (1,3,5-tris(*N*-phenylbenzimidazol-2-yl)-benzene (TPBi)), but also used as efficient emissive materials with low-efficiency roll-off.^26–36^ Apart from fused imidazole-based emissive materials, pyrene-substituted imidazole blue emitters show excellent efficiency.^37,38^ Similar to the TADF mechanism, the CT excited-state component in the HLCT excited-state facilitates the reverse intersystem crossing (RISC) process by small Δ*E*_ST_, leading to high external utilization efficiency (EUE). In contrast to TADF emitters, HLCT emitters exhibit higher efficiency with short excited lifetime. In some blue emissive weakly coupled HLCT materials, the emissive excited state possesses a pure locally excited (LE) state rather than a CT excited state and it is advantageous for blue-shifted emission with high efficiency.^39,40^

Herein, we report blue HLCT materials, namely, 2-(4-(10-(9*H*-carbazol-9-yl) anthracen-9-yl)phenyl)-1-(naphthalen-1-yl)-1*H*-phenanthro[9,10-*d*]imidazole (CAPI) and 4-(2-(4-(10-(9*H*-carbazol-9-yl)anthracen-9-yl)phenyl)-1*H*-phenanthro[9,10-*d*]imidazol-1-yl) naphthalene-1-carbonitrile (CCAPI), consisting of carbazole as the donor and phenanthroimidazole as the acceptor with anthracene spacers. The anthracene moiety increases the conjugation length, leading to an enhanced % LE component, and these materials show excellent performances with small efficiency roll-off. The analyses of single-carrier devices revealed that these compounds have good bipolar carrier transport characteristics and non-doped blue devices with CCAPI as the emitting layer showed maximum external quantum efficiency (EQE) of 10.5% and exciton utilisation efficiency (EUE) of 83%. The small energy splitting (Δ*E*_ST_ ≈ 0) promotes the RISC process, and the dark triplet excitons are effectively converted into singlet excitons and enhance the efficiency.

## 2 Experimental section

### 2.1. Synthesis of emissive materials

The chemicals used in the experimental section were obtained from Sigma-Aldrich. The synthetic routes of the emissive materials are outlined in Scheme S1.†

#### (a) Synthesis of bromophenanthroimidazoles (BrPPI/CNBrPPI)

A mixture of 9,10-phenanthraquinone (9.61 mmol), naphthalen-1-amine (BrPPI)/4-aminonaphthalene-1-carbonitrile (CNBrPPI) (38.42 mmol), 4-bromobenzaldehyde (9.61 mmol) and ammonium acetate (48.03 mmol) in acetic acid (30 ml) was refluxed for 12 h (120 °C; N_2_ stream). The solution was poured into ethanol and the separated solid was dried and purified by column chromatography. A pure yellow sample was collected and used for synthesizing blue-emissive materials.

#### (b) 2-(4-Bromophenyl)-1-(naphthalene-1-yl)-1*H*-phenanthro[9,10-*d*]imidazole (BrPPI)

Yield: 80%. ^1^H NMR: 7.30–7.36 (m, 6H), 7.48 (d, 2H), 7.69 (t, 3H), 7.82–7.88 (m, 4H), 8.12 (d, 2H), 8.93 (d, 2H).^13^C NMR: 122.3, 123.2, 124.2, 126.3, 126.7, 127.3, 127.7, 127.9, 128.3, 129.8, 130.8, 132.3, 134.7, 149.5. MS: *m*/*z*. 498.08 [M^+^]; calcd 497.54.

#### (c) 4-(2-(4-Bromophenyl)-1*H*-phenanthro[9,10-*d*]imidazol-1-yl)naphthalene-1-carbonitrile (CN BrPPI)

Yield: 85%. ^1^H NMR: 7.37–7.61 (m, 7H), 7.80–7.88 (m, 6H), 8.12–8.22 (m, 3H), 8.93 (d, 2H). ^13^C NMR: 109.3, 115.9, 121.6, 122.3, 123.7, 126.7, 127.4, 127.7, 127.9, 128.4, 128.6, 129.6, 131.4, 132.1, 133.3, 136.0, 149.4. MS: *m*/*z*. 523.09 [M^+^]; calcd 521.89.

### 2.2. Synthesis of dioxaboranylphenylphenanthroimidazoles (PPIB/CNPPIB)

A mixture of BrPPI (PPIB)/CNBrPPI (CNPPIB) (6.53 mmol), bis(pinacolato)diboron (7.84 mmol), Pd(dppf)Cl_2_ (0.13 mmol) and KOAc (19.60 mmol) in 1,4-dioxane (30 ml) was refluxed for 48 h (N_2_ stream; 90 °C). The resulting solution was extracted with dichloromethane and the separated solid was purified by column chromatography to obtain a light yellow powder.

#### (a) 2-(4-(4,4,5,5-Tetramethyl-1, 3,2-dioxaborolan-2-yl)phenyl)-1-(naphthalene-1-yl)-1*H*-phenanthro[9,10-*d*]imidazole (PPIB)

Yield: 72%. ^1^H NMR: 1.28 (d, 12H), 7.30 (m, 6H), 7.50 (d, 2H), 7.7 (t, 3H), 7.82–7.88 (m, 4H), 8.12 (d, 2H), 8.93 (d, 2H). ^13^C NMR: 21.1, 83.3, 122.3, 124.1, 126.4, 126.7, 127.4, 127.8, 127.7, 128.2, 130.7, 131.5, 132.4, 134.5, 134.7, 149.3. MS: *m*/*z*. 496.20 [M^+^]; calcd 495.38.

#### (b) 4-(2-(4-(4,4,5,5-Tetramethyl-1,3,2-dioxaborolan-2-yl)phenyl)1*H*-phenanthro[9,10-*d*]imidazol-1-yl)naphthalene-1-carbonitrile (CNPPIB)

Yield: 75%. ^1^H NMR: 1.25 (d, 12H), 7.30 (2, 2H), 7.50–7.60 (m, 5H), 7.80–7.88 (m, 6H), 8.12–8.20 (m, 3H), 8.93 (d, 2H). ^13^C NMR: 21.2, 83.3, 109.3, 115.7, 121.6, 122.5, 123.9, 126.6, 126.7, 127.6, 127.8, 128.2, 128.6, 130.5, 131.4, 132.6, 134.5, 136.2, 149.2. MS: *m*/*z*. 521.24 [M^+^]; calcd 519.78.

### 2.3. Synthesis of bromoanthracenylphenanthroimidazoles (BrPIAN/BrCNPIAN)

A mixture of PPIB (BrPIAN)/CNPPIB (BrCNPIAN) (5.35 mmol) and 9,10-dibromoanthracene (4.46 mmol) was refluxed with K_2_CO_3_ (6.51 mmol) and Pd(PPh_3_)_4_ (0.130 mmol) in tetrahydrofuran : water (50 : 10 ml) at 70 °C (N_2_ stream; 48 h). The yellow-coloured solution was poured into ethanol and the solid was purified by the conventional method.

#### (a) 2-(4-(10-Bromoanthracen-9-yl)phenyl)-1-(naphthalen-1-yl)-1*H*-phenanthro[9,10-*d*]imidazole (BrPIAN)

Yield: 65%.^1^H NMR: 7.30–7.41 (m, 8H), 7.54 (m, 4H), 7.69–7.70 (m, 5H), 7.82–7.92 (m, 6H), 8.12 (d, 2H), 8.93 (d, 2H).^13^C NMR: 121.6, 122.5, 124.2, 126.4, 126.6, 126.7, 127.4, 127.6, 127.8, 128.3, 128.5, 129.5, 129.7, 131.2, 132.3, 134.4, 136.6, 149.3. MS: *m*/*z*. 674.10 [M^+^]; calcd 675.35.

#### (b) 4-(2-(4-(10-Bromoanthracen-9-yl)phenyl)-1*H*-phenanthro[9,10-*d*]imidazol-1-yl) naphthalene-1-carbonitrile(BrCNPIAN)

Yield: 68%. ^1^H NMR: 7.37–7.41 (m, 5H), 7.54–7.60 (m, 8H), 7.80–7.92 (m, 8H), 8.12–8.20 (m, 3H), 8.93 (d, 2H).^13^C NMR: 109.5, 115.5, 121.8, 122.5, 123.9, 126.4, 126.9, 126.6, 127.4, 127.6, 128.1, 129.8, 129.6, 131.2, 132.4, 133.3, 134.4, 136.1, 136.7, 149.5. MS: *m*/*z*. 699.14 [M^+^]; calcd 700.32.

### 2.4. Synthesis of emissive materials (CAPI/CCAPI)

The blue emissive materials, CAPI/CCAPI were synthesized through a Suzuki coupling reaction of BrPIAN/BrCNPIAN with carbazole. A solution of BrPIAN (CAPI)/BrCNPIAN (CCAPI) (1.95 mmol), 9*H*-carbazole (4.29 mmol), Pd(PPh_3_)_4_ (0.20 mmol) and aqueous K_2_CO_3_ (2 M, 6 ml) in tetrahydrofuran : water (50 : 10 ml) was refluxed at 70 °C (48 h; N_2_ atmosphere). The reaction solution was extracted with dichloromethane and the separated solid was purified by column chromatography.

#### (a) 2-(4-(10-(9*H*-Carbazol-9-yl)anthracen-9-yl)phenyl)-1-(naphthalen-1-yl)-1*H*-phenanthro[9,10-*d*]imidazole (CAPI)

Yield: 66%; mp 266 °C; ^1^H NMR: 7.01–7.10 (m, 4H), 7.30–7.38 (m, 6H), 7.39 (m, 4H), 7.55 (d, 6H), 7.68–7.67 (m, 7H), 7.80–7.87 (m, 4H), 8.10 (d, 2H), 8.95 (d, 2H) ^13^C NMR: 111.1, 119.0, 120.1, 121.0, 122.2, 124.1, 126.3, 126.4, 126.5, 127.2, 127.3, 127.8, 128.0, 128.3, 128.4, 129.6, 129.8, 130.7, 131.5, 131.6, 134.6, 136.6, 138.8, 139.7, 149.4. FT-IR (KBr), *ν* (cm^−1^): 3065, 3053, 3146, 3092, 3034, 1598, 1518. MS: *m*/*z*. 761.28. [M^+^]; calcd 761.91.

#### (b) 4-(2-(4-(10-(9*H*-Carbazol-9-yl)anthracen-9-yl)phenyl)-1*H*-phenanthro[9,10-*d*]imidazol-1-yl)naphthalene-1-carbonitrile (CCAPI)

Yield: 69%; mp 272 °C. ^1^H NMR: 7.03–7.09 (m, 4H), 7.27–7.32 (m, 4H), 7.43 (m, 3H), 7.52–7.61 (m, 6H), 7.54 (d, 2H), 7.65–7.69 (m, 4H), 7.79–7.88 (m, 6H), 8.10–8.18 (m, 3H), 8.91 (d, 2H). ^13^C NMR: (109.5, 111.1, 115.8, 119.0, 120.1, 121.7, 122.2, 122.4, 123.8, 126.3, 126.5, 127.3, 127.5, 128.3, 128.4, 128.5, 129.6, 129.8, 131.5, 131.6, 133.4, 136.0, 136.5, 138.8, 139.7, 149.4. FT-IR (KBr), *ν* (cm^−1^): 3058, 3043, 3032, 2974, 2867, 1595, 1523, 1487, 1367. MS: *m*/*z*. 785.28 [M^+^]; calcd 786.92.

## 3 Results and discussion

The synthetic route for the emissive materials is displayed in Scheme S1.† Efficient blue emitters, namely, 2-(4-(10-(9*H*-carbazol-9-yl)anthracen-9-yl)phenyl)-1-(naphthalen-1-yl)-1*H*-phenanthro[9,10-*d*]imidazole (CAPI) and 4-(2-(4-(10-(9*H*-carbazol-9-yl)anthracen-9-yl)phenyl)-1*H*-phenanthro[9,10-*d*]imidazol-1-yl)naphthalene-1-carbonitrile (CCAPI) were synthesized by a Suzuki-coupling reaction with substantial yield, and the formation of CAPI and CCAPI was confirmed by spectral techniques.

### 3.1. Thermal and electrochemical studies

**Fig. 1:**
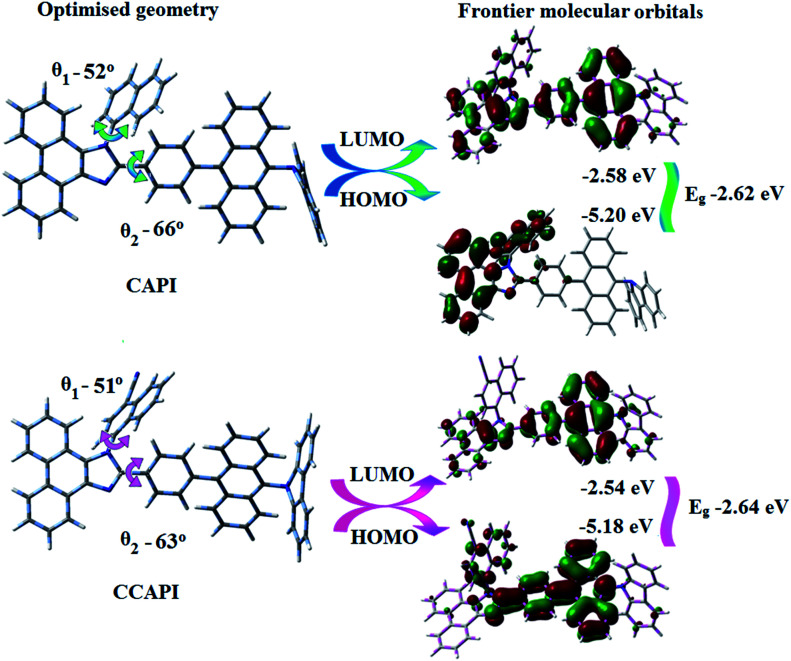
Optimised geometry with dihedral angles and frontier molecular orbitals of CAPI and CCAPI.

**Fig. 2:**
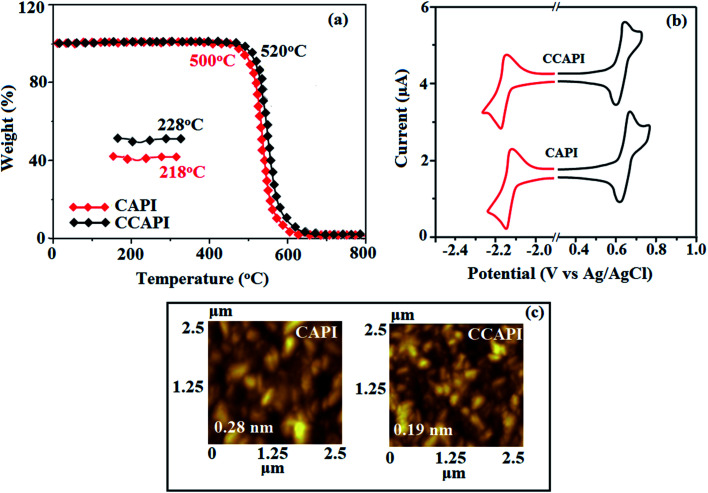
(a) TGA and DSC graphs of CAPI and CCAPI. (b) Cyclic voltammogram and (c) AFM images of CAPI and CCAPI.

The twisted molecular architecture of CAPI and CCAPI, having a twist angle of ∼52° between the styryl substituent (C2) and the phenanthrimidazole core, effectively suppressed the conjugation and intermolecular π–π stacking (Fig. 1). The twisted molecular architecture increased the thermal stability of CAPI (*T*_g_/*T*_d_: 218/500 °C) and CCAPI (*T*_g_/*T*_d_: 228/528 °C) (Fig. 2). Because of the C/N-side coupling with bulky substituents, the rigid phenanthroimidazole showed high glass transition temperature and interaction of substituents at C2 with N1 of the phenanthroimidazole core induced more condensed molecular packing. The higher *T*_g_ and *T*_d_ are essential for applications in devices. The morphological stability of these compounds was examined by atomic force microscopy at 30 °C and 90 °C for 12 h (Fig. 2). The root-mean-square [CAPI – 0.28 nm; CCAPI – 0.19 nm] analysis showed the absence of remarkable surface modification before and after annealing, which further supports the suitability of these materials for fabrication. CAPI and CCAPI showed a redox process with an oxidation onset potential (*E*_onset_) of 0.63 V (CAPI) and 0.61 V (CCAPI), which revealed that these bipolar carrier transporting materials are electrochemically stable (Fig. 2). The HOMO = −(*E*_ox_*vs.* Ag/Ag^+^ − *E*_1/2_^+^*vs.* Ag/Ag^+^ + 4.8) eV/LUMO = −(*E*_red_*vs.* Ag/Ag^+^ − *E*_1/2_^−^*vs.* Ag/Ag^+^ + 4.8) eV have been calculated as CAPI (−5.20/−2.58 eV) and CCAPI (−5.18/−2.54 eV).

### 3.2. Photophysical studies

**Fig. 3:**
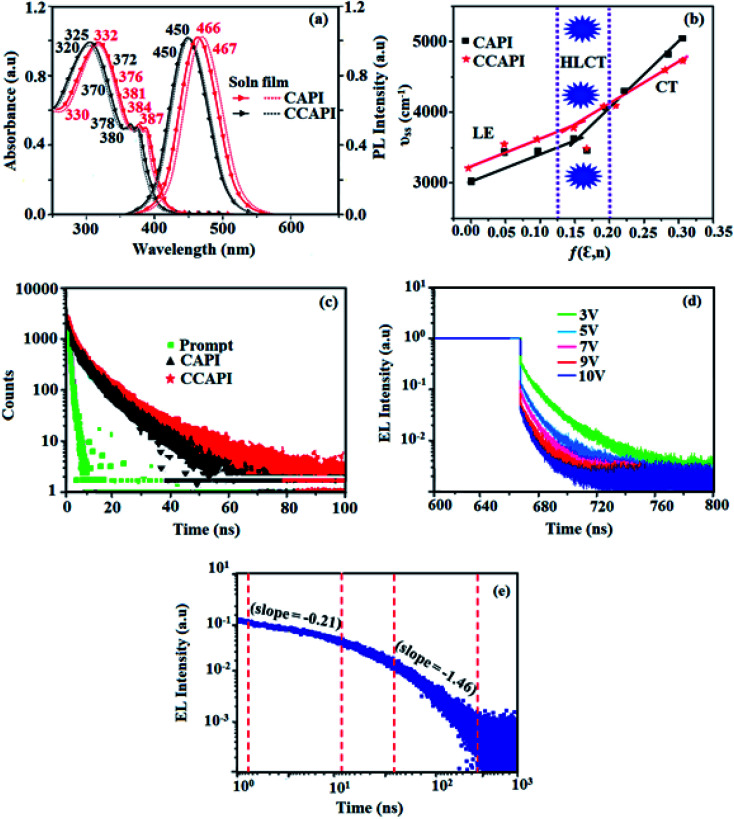
(a) Normalised absorption and emission spectra; (b) Lippert-Mataga plot; (c) lifetime decay of CAPI and CCAPI; (d) transient EL decay of CCAPI at different voltages and (e) amplified EL decay of the delayed component of CCAPI.

The absorption of emissive materials has been studied with various solvents (Fig. S1, Tables S1 and S2†) and in the film state (Fig. 3 and Table 1). The absorption in the region of 320–332 nm is attributed to the π–π* (LE) transition of phenanthroimidazole and carbazole moieties,^41,42^ whereas the absorption at around 370 nm is ascribed to the π–π* transition of the anthracene moiety.^43^ The transition around 380 nm is due to the CT transition [(*λ*_ab_ (sol/film): CAPI: 332, 384, 387/330, 376, 381 nm; CCAPI: 325, 372, 380/320, 370, 378 nm]. The high extinction coefficient (*ε*_max_) of D–π–A molecule is due to increase of conjugation length and confirm CT transition from donor to acceptor:^46,47^ [CAPI: 332 nm (*ε*_max_ – 30120.48 cm^−1^ M^−1^), 384 nm (*ε*_max_ – 26 041.67 cm^−1^ M^−1^); 387 nm (*ε*_max_ – 25 839.79 cm^−1^ M^−1^); CCAPI: 325 nm (*ε*_max_ −30 769.23 cm^−1^ M^−1^), 375 nm (*ε*_max_ – 26 666.67 cm^−1^ M^−1^); 380 nm (*ε*_max_ – 26 315.79 cm^−1^ M^−1^)].^44,45^ The small shift in the film state shows the existence of weak π–π* intermolecular stacking.^48^ The UV absorption spectra of CAPI and CCAPI changed shape and position with increasing solvent polarity due to small dipole moment changes at the ground state in different solvents (Fig. S1†). The photoluminescence characteristics (*λ*_emi_ (sol/film)) of these compounds were studied in film and various solvents (Fig. S1†) [CAPI: 466/467; CCAPI: 450/450 nm] (Fig. 3 and Table 1). The solvatochromic emission spectra of CAPI and CCAPI from higher polarity solvents were remarkably broad and red-shifted from *n*-hexane to acetonitrile due to twisting by 9,10-substituted anthracene, and were further supported by natural transition orbitals (Fig. S2 – CAPI and Fig. S3 – CCAPI†).

**Table 1:** Optical and thermal properties of CAPI and CCAPI

Emitters	CAPI	CCAPI
*λ*_ab_ (nm) (sol/film)	332, 384,387/330,376,381	325 372 380/320 370 378
*λ*_em_ (nm) (sol/film)	466/467	450/450
*T*_g_/*T*_d_ (°C)	218/500	228/520
*ϕ* (soln/film)	80/50	88/62
HOMO/LUMO (eV)	−5.20/−2.58	−5.18/−2.54
*E*_g_ (eV)	−2.62	−2.64
*τ* (ns)	2.38	2.49
*k*_r_ × 10^8^ (s^−1^)	0.33	0.35
*k*_nr_ × 10^8^ (s^−1^)	0.09	0.05

The interaction of the dipole moment of the solute with the solvent have been analysed by the Lippert-Mataga model:^49^*hc*(*ε̃*_abs_ − *ε̃*_flu_) = *hc*(*hcε̃*^vac^_abs_ − *hcε̃*^vac^_flu_) + 2(*μ*_e_ − *μ*_g_)^2^/*a*_o_^3^*f*(*ε*,*n*) [*f*(*ε*,*n*): [(*ε* − 1/2 *ε* + 1) − 1/2 (*n*^2^ − 1/2*n*^2^ + 1)]; [*μ*_g_ (ground state dipole moment), *μ*_e_ (excited state dipole moment), *f* (orientation polarizability), *ε̃*_abs_ (absorption maximum), *ε̃*^vac^_abs_ (absorption maximum extrapolated to gas phase), *ε̃*_flu_ (fluorescence maximum), *ε̃*^vac^_flu_ (fluorescence maximum extrapolated to gas-phase), *a*_o_ (Onsager cavity), *ε* (solvent dielectric constant) and *n* (solvent refractive index)] (Fig. 3). The emission spectra gradually broadened and showed less structure with larger red-shifts, which supports that the excited state has a strong CT component. The red-shifted emission is due to the twisted conformation of these emitters, which leads to easier charge transfer from donor to acceptor *via* anthracene linker.

The two-section linear relation observed from the Lippert-Mataga plot (Fig. 3b) revealed that a line with a small slope in solvents with *f* ≤ 0.1 is due to the LE-like excited state component with a lower dipole moment (CAPI – 7.32 D and CCAPI – 5.00: DFT calculation: CAPI – 8.3 D and CCAPI – 7.5 D), whereas the higher slope in solvents with *f* ≥ 0.2 is because of the CT-like excited state component with a greater excited state dipole moment (CAPI – 24.3 D and CCAPI – 25.2 D).^50^ This highlights the coexistence and hybridization of the LE and CT excited state components (CAPI – 22.5 D and CCAPI – 23.4 D) [triphenylamine derivative TPAAnPI showed 9.9 D and 25.3 D].^51^ The higher dipole moment corresponding to the CT-region is greater than that of the CT molecule 4-(*N*,*N*-dimethylamino)benzonitrile (DMABN, 23 D), whereas the lower dipole moment in the LE-region is a little higher than those of conventional LE molecules such as anthracene and PI, indicating that CAPI and CCAPI are HLCT materials.

The solvatochromic data of these compounds were fitted with two straight lines corresponding to two different dipole moments because of the coexistence and hybridization of the LE and CT excited state components.^50^ The transformation of the slope between ether (*f* = 0.10) and ethyl acetate (*f* = 0.20) implies that CAPI and CCAPI have HLCT emissive states (intercrossed excited state of LE and CT): high % CT contribution in solvents with *f* ≥ 0.2, % LE dominates in solvents with *f* ≤ 0.1 and mixed contribution of LE and CT in moderate polarity solvents. The new blue emitters show high PLQY (soln/film) of CAPI (80/50) and CCAPI (88/62) and high PLQY is essential for efficient blue OLEDs (Table 1). It is unique that the CT material shows efficient deep-blue emission; the co-emission from LE and CT (intercrossed CT and LE state) is likely to be the reason for the high fluorescence yield. The high *f* (oscillator strength: *λ*_abs_/*λ*_emi_) in CHCl_3_ relative to the gas phase for CAPI [gas: 362 (*f* − 1.3687)/434 (*f* − 1.736): CHCl_3_-384 (*f* − 1.8923)/466 (*f* − 2.3098)] and CCAPI [gas: 356 (*f* − 1.4167)/433 (*f* − 1.5098): CHCl_3_-372 (*f* − 1.8932)/450 (*f* − 2.4531)] implies that higher luminescence will be obtained from the intercrossed excited state. These materials demonstrate one nanosecond lifetime without any delayed components (Fig. 3) and the energy gap of the singlet and triplet energy levels of CAPI and CCAPI is estimated to be less than 0.1 eV, which is also a feature of typical HLCT materials.

In low-polarity solvents, the PLQYs of CAPI/CCAPI remain unchanged (0.60/0.66 − *n*-hexane, 0.65/0.68 – isopropyl ether), which implies that the LE dominated emission from the low lying S_1_ state. In a medium-polarity solvent (THF), a high PLQY of 0.80/0.88 for CAPI/CCAPI was obtained due to the hybridization of LE with the CT excited state. In a high-polarity solvent (acetonitrile), a decrease in PLQY of (0.63/0.67) for CAPI/CCAPI was obtained, which is similar to that in low polarity solvents. As a result, the PLQY in solvents and film are satisfactory for OLED fabrication. The HLCT character can also be confirmed by the mono-exponential, nanosecond lifetimes of CAPI and CCAPI in solution (1.38/1.49 ns – *n*-hexane, 1.25/1.29 ns – ether, 2.38/2.49 ns – THF, 3.56/3.85 ns – acetonitrile) (Fig. 3) and show high radiative transition rates, CAPI (*k*_r_/*k*_nr_): 0.43/0.29 (*n*-hexane); 0.51/0.29 (ether); 0.33/0.09 (THF); 0.17/0.11 – acetonitrile and CCAPI (*k*_r_/*k*_nr_): 0.44/0.23 (*n*-hexane); 0.52/0.25 (ether); 0.35/0.05 (THF); 0.17/0.08 (acetonitrile) (Table 2). The CAPI/CCAPI oscillator strength (0.6089/0.6636) in the S_1_ excited state is consistent with PLQY in low polarity solutions. The excited state (S_1_) dipole moments of CAPI and CCAPI of 7.32 D and 5.00 D are also in accordance with the experimental results. Therefore, the emissive state of CAPI/CCAPI belongs to the S_1_ excited state and serves as exciton utilization channel. The S_0_ excited state exhibited obvious CT character according to NTO analysis. The NTO for S_0_ → S_3_ (CAPI) and S_0_ → S_4_ (CCAPI) transition displays a total CT transition character with hole distribution on the carbazole moiety and particle distribution on anthracene with minor overlap on the adjacent moiety, and T_13_ and T_4_ are the corresponding triplet CT excited states for CAPI (*E*_S_3_-T_13__ – 0.00 eV) and CCAPI (*E*_S_4_-T_8__ – 0.00 eV), respectively. Therefore, this could increase the RISC between S_3_ and T_13_ (CAPI) and S_3_ and T_4_ (CCAPI) excited states and enhance the exciton utilisation efficiency [% EUE: CAPI/CCAPI – 64/83: ^b^70/95].

**Table 2:** Radiative and non-radiative constants for CAPI and CCAPI with different solvents

Solvents	CAPI				CCAPI			
	PLQY (%)	*τ* (ns)	*k*_r_ (10^9^) s^−1^	*k*_nr_ (10^9^) s^−1^	PLQY (%)	*τ* (ns)	*k*_r_ (10^9^) s^−1^	*k*_nr_ (10^9^) s^−1^
Hexane	60.0	1.38	0.43	0.29	66.0	1.49	0.44	0.23
Ether	64.8	1.25	0.51	0.29	68.0	1.29	0.52	0.25
THF	80.0	2.38	0.33	0.09	88.0	2.49	0.35	0.05
Acetonitrile	62.6	3.56	0.17	0.11	67.3	3.85	0.17	0.08

### 3.3. Theoretical calculation

To gain more insight into the analysis of the HLCT emissive state, molecular optimization and frontier molecular orbital (FMO) analysis for CAPI and CCAPI were carried out (DFT/B3LYP/6-31G (d, p)) (Fig. 1). The blue emitters CAPI and CCAPI consist of the 9,10-substituted anthracene moiety involving the twisted D–π–A molecular configuration with torsional angles of 52° and 51° for N-side coupling, and 66° and 63° for C-side coupling, respectively. These carbazole derivatives show higher C-coupling torsional angles but smaller N-coupling angles as compared with triphenylamine derivatives TPAAnPI (torsional angles of 53.7°-C-side) and 69.0°-N-side).^51^

Therefore, the calculated FMO exhibit partially separated characteristics. The highest occupied molecular orbital (HOMO) of CAPI is mainly localized on the phenanthrimidazole core and the LUMO is localized on the phenyl, anthracene and partially on phenanthrimidazole. In CCAPI, the HOMO is distributed on anthracene and the LUMO is fully distributed on the anthracene moiety, suggesting that the HOMO → LUMO transition involves an intercrossed CT and π–π* transition character, reflecting HLCT character. Furthermore, NTO analyses were performed for the singlet and triplet excited states based on the S_0_ state geometry using the time-dependent DFT (TD-DFT) method at the same level as S_0_. The S_0_ → S_1_ and S_0_ → S_2_ transitions of CAPI and CCAPI are the radiative π–π* and non-radiative n–π* transitions of anthracene, respectively, which efficiently increased the RISC between the S_3_ and T_13_ excited states (CAPI) and the S_4_ and T_8_ (CCAPI) excited states, and enhanced the exciton utilization efficiency.

The HONTOs and LUNTOs of CAPI and CCAPI (Fig. S2, S3, Tables S3 and S4†) exhibited hybrid splitting state character from the interstate coupling of LE with CT levels. The interstate hybridization coupling of LE with the CT state wave function is given by *Ψ*_S_1_/S_2__ = *c*_LE_*Ψ*_LE_ ± *c*_CT_*Ψ*_CT_. The % CT of these emitters increased with steric hindrance with increasing aromatic substituent size and the increase in % LE in the S_1_ state resulted in higher photoluminescence efficiency (*η*_PL_). The single emissive state of CAPI and CCAPI has been investigated by the excitation energies of the LE and CT states. A large energy gap (Δ*E*_ST_) between T_13_ and T_1_ of CAPI (1.89 eV) and T_8_ and T_1_ of CCAPI (1.08 eV) arose from phenanthroimidazole acceptor group.^50,51^ A very small Δ*E*_ST_ between S_3_ and T_13_ (0.00 eV – CAPI) and S_4_ and T_8_ (0.00 eV – CCAPI) states facilitated the RISC (*T*_*n*_ → S_1_) process due to HLCT character (the S_3_–T_12_ energy gap of the triphenylamine derivative TPAAnPPI is only 0.0013 eV).^51^

CAPI and CCAPI show high photoluminescence efficiency (*η*_PL_), high exciton utilisation efficiency (EUE) and high external quantum efficiency (EQE) because of the increased LE component in the S_1_ state. The small Δ*E*_ST_ of these materials arises from spatially separated HOMO and LUMO.^46^

Similar hole–electron wave functions between the singlet excited states in CAPI and CCAPI indicate the non-equivalent hybridization of the initial LE with the CT state (CAPI and CCAPI). The degree of hybridization in CAPI and CCAPI depends on both the initial Δ*E*_LE,CT_ and the coupling strength.^54^ In the transition density matrix (TDM) plot (Fig. S4 – CAPI and Fig. S5 – CCAPI†), the diagonal parts represent the LE component and the off-diagonal zone represents the CT component. Depending on electronic coupling, electrons are transferred from donor to acceptor on excitation, which was studied *via* the electron density distribution in both the ground and excited states. The computed distance between holes and electrons, H as well as *t* indexes and the RMSD of electrons and holes of these emitters are shown in Tables S5–S12.†

**Fig. 4:**
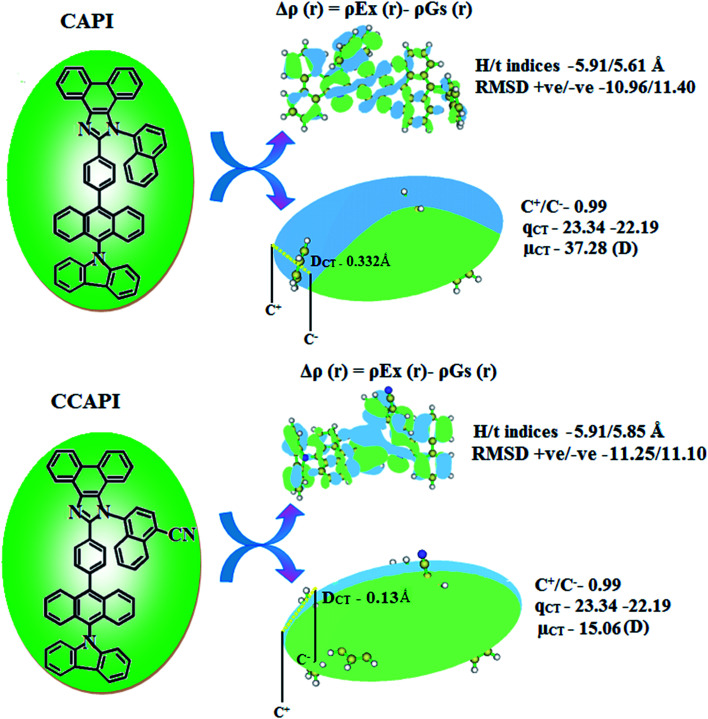
Graphical representation of *D*_CT_ and centroid of charges [*C*+(*r*)/*C*−(*r*); isosurface for CAPI and for CCAPI (0.1 au)].

The formation of HLCT is supported by the Δ*r* index (average of the hole–electron distance (*d*_h^+^–*e*^−^_): *r* < 2.0 Å LE; Δ*r* > 2.0 Å CT) and indicates the nature of excitation (LE or CT) (Tables S13 and S14†). The LE (valence excitation) is associated with short distances (*d*_h^+^–*e*^−^_), whereas CT excitation is related to larger distances (*d*_h^+^–*e*^−^_). The dark triplet exciton is harvested through the RISC process by a hot CT mechanism in the electroluminescence process without delayed emission and leads to high exciton utilization (*η*_s_) in CAPI and CCAPI like phosphorescent materials.^44,45^ The increasing % LE component and hybridization of LE with CT components result in high *η*_PL_, and high *η*_s_ leads to enhanced device performances (Table 3). The computed overlap of the condensed function (*ρ*^+^ and *ρ*^−^) in CAPI and CCAPI is 0.99 (Fig. 4 and Table S15†), and the *H*/*t* index for CAPI and CCAPI are 5.91/5.61 and 5.91/5.85 Å, respectively. The CT index (*D*_CT_ − *H* index) is another measure of the hole–electron separation (eqn S15 and S16†), and the calculated *D*_CT_/*μ*_CT_ of CAPI (0.33/37.28) and CCAPI (0.13/15.06) further confirmed the HLCT formation. A non-zero *t* index implies the severe overlap of holes with electrons and the Eigenvalue (>0.98) confirmed the hybridization with predominant excitation pairs (94% of transition).

**Table 3:** Electroluminescent efficiencies of CAPI and CCAPI

Emitters	*V*_1000_ (V)	*η*_c_ (cd A^−1^)	*η*_p_ (lm W^−1^)	EQE_max_ (%)	*L* (cd m^−2^)	EL (nm)	CIE (*x*,*y*)	*η*_roll-off_ (%)	*η*_s_ (%)
CAPI^a^	3.00	13.32	13.00	8.40	26 490	467	0.15,0.18	1.07	64
CAPI^b^	2.90	14.06	14.81	8.89	28 801	467	0.15,0.18	1.02	70
CCAPI^a^	2.87	15.26	13.89	10.50	30 628	450	0.15,0.20	4.76	83
CCAPI^b^	2.80	16.83	15.32	12.00	32 546	450	0.15,0.20	0.83	95

a ITO/PEDOT:PSS (40 nm)/NPB (5 nm)/TCTA (30 nm)/CAPI or CCAPI (20 nm)/TPBi (30 nm)/LiF (1 nm)/Al (100 nm).

b ITO/PEDOT:PSS (40 nm)/TCTA (30 nm)/CAPI or CCAPI (20 nm)/TPBi (30 nm)/LiF (1 nm)/Al (100 nm).

Fig. S6† shows the potential energy surface (PES) scan of CAPI and CCAPI in the gas phase and different polarity solvents. In the gas phase, the S_3_ (CAPI) and S_4_ (CCAPI) states are not mixed with the S_1_ state due to a large Δ*E*_S_3_-S_1__ (CAPI) and Δ*E*_S_4_-S_1__ (CCAPI). In low polarity solvents, the S_3_ (CAPI) and S_4_ (CCAPI) states crossed the S1 state, whereas in high polarity solvents, *E*_S_3__ (CAPI) and *E*_S_4__ (CCAPI) decreased sharply and became the lowest excited state. The energetic closeness in moderate-polarity solvents leads to the enhanced mixing of S_3_ (CAPI) and S_4_ (CCAPI) with S_1_ (the larger dipole moments of the S_3_ (CAPI) and S_4_ (CCAPI) states lead to stabilization in high polarity solvents). Therefore, the S_1_ state is dominated by LE character in low polarity medium; the S_1_ state is dominated by mixing the LE and CT character in moderate polarity medium and the S_1_ state is dominated by CT character in high polarity medium.

### 3.4. Electroluminescent properties

**Fig. 5:**
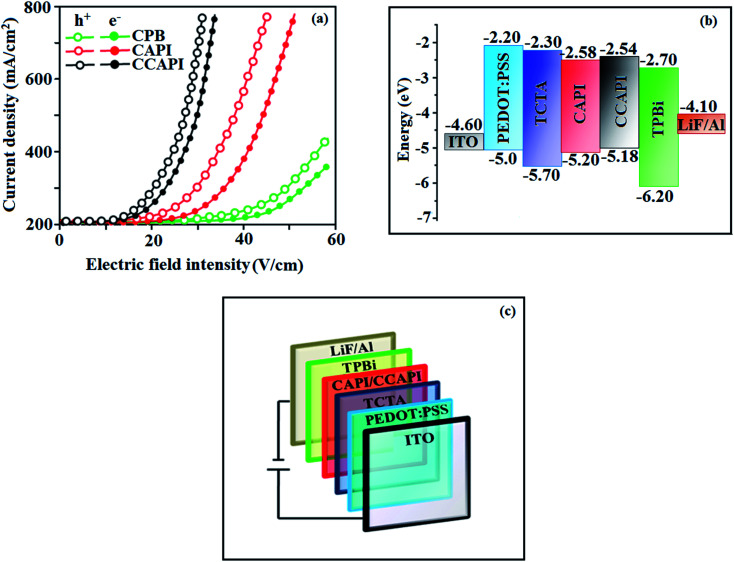
(a) Single-carrier devices: current density *vs.* electric field intensity; (b) energy level diagram of non-doped devices and (c) configuration of non-doped devices.

To understand the carrier transport abilities of CAPI and CCAPI, hole-only (HOD) and electron-only (EOD) devices have been fabricated with the configuration of ITO/NPB (8 nm)/CAPI or CCAPI (40 nm)/NPB (8 nm)/Al (100 nm) (hole-only device): NPB (LUMO: −2.3 eV) in the hole-only device can prevent electron injection from the Al cathode (*E*_f_ – 4.3 eV)^47^ and ITO/TPBi (8 nm)/CAPI or CCAPI (40 nm)/TPBi (8 nm)/LiF (1 nm)/Al (100 nm) (electron-only device): TPBi (HOMO: −6.2 eV) in the electron-only device with ITO (*E*_f_ – 4.8 eV) anode can prevent hole injection.^52^ The current densities *versus* voltage characteristics of hole-only and electron-only devices revealed that these compounds are bipolar materials (Fig. 5). The higher electron current density of CAPI and CCAPI based devices relative to the CBP device revealed that these bipolar materials transport electrons as well as holes effectively than CBP.^55–57^

To explore the potential application of CAPI and CCAPI as fluorescent materials, non-doped blue fluorescent OLEDs have been fabricated with the configuration of ITO/PEDOT:PSS (40 nm)/NPB (5 nm)/CAPI or CCAPI (20 nm)/TPBi (30 nm)/LiF(1 nm)/Al (100 nm) (Fig. 5) and ITO/PEDOT:PSS (40 nm)/TCTA (30 nm)/CAPI or CCAPI (20 nm)/TPBi (30 nm)/LiF (1 nm)/Al 100 nm) (Fig. S7†) [polyethylenedioxythiophene/polystyrene sulfonate (PEDOT : PSS) – hole injecting layer; (*N*,*N*′-diphenyl-*N*,*N*′-bis(1-naphtyl)-1,1′-biphenyl-4,4′′-diamine) (NPB) – hole transporting layer; 4,4′,4′′-tri(*N*-carbazolyl) triphenylamine (TCTA) – hole transporting and electron blocking layer; 1,3,5-tri(phenyl-2benzimidazolyl)benzene (TPBi) – electron transporting and hole blocking layer; LiF – electron injecting layer].

**Fig. 6:**
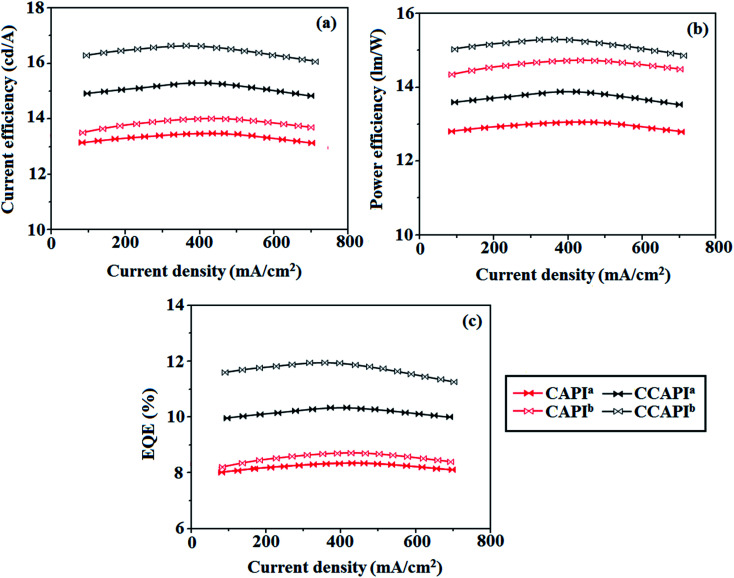
Device efficiencies: (a) current efficiency − current density; (b) power efficiency − current density and (c) external quantum efficiency – current density.

**Fig. 7:**
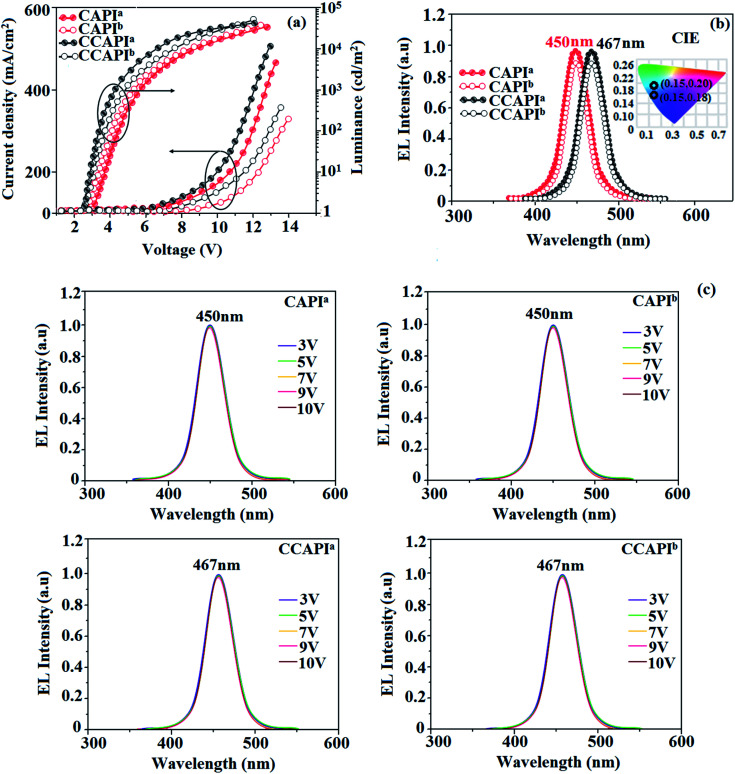
Device efficiencies: (a) luminance – current density – voltage; (b) EL spectra (inset: CIE coordinates) and (c) normalized EL specta at different voltages.

**Fig. 8:**
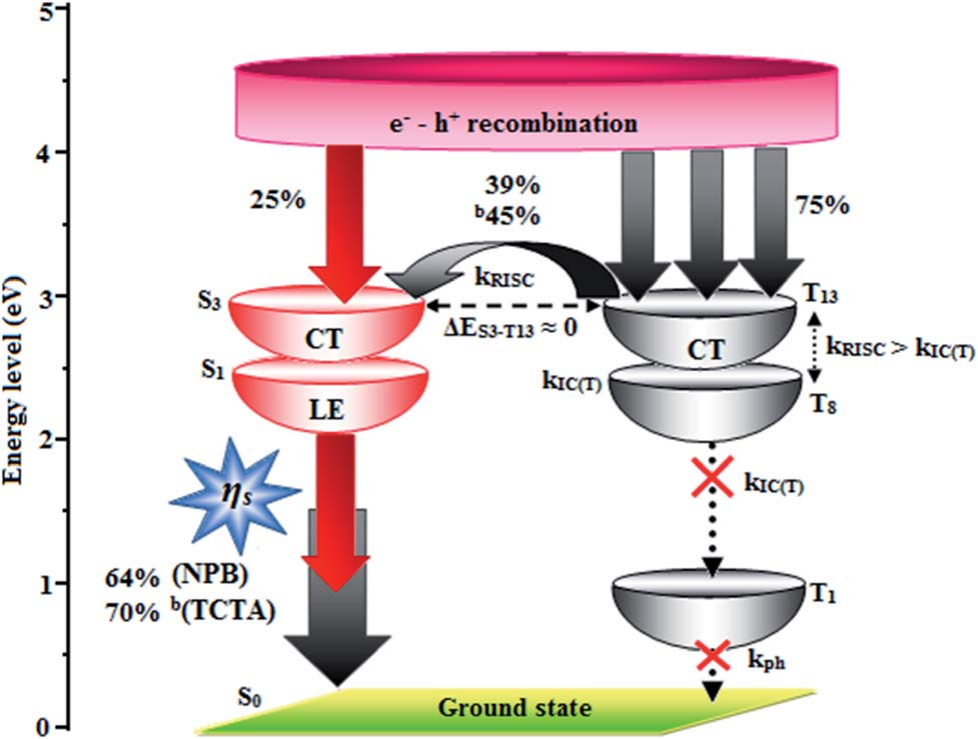
Scheme of hole and electron recombination in OLEDs of CAPI.

The non-doped device based on CAPI/CCAPI showed blue emission (467/450 nm) with CIE of (0.15, 0.18)/(0.15, 0.20) and exhibited maximum current efficiency (*η*_c_), power efficiency (*η*_p_) and EQE of 13.32/15.26 cd A; 13.0/13.89 lm W^−1^; 8.4/10.5% with luminance of 26 490/30 628 cd m^−2^, respectively (Fig. 6, 7 and Table 2). The external quantum efficiency [*η*_EQE_ = *η*_out_ × *η*_rc_ × *η*_γ_ × *Φ*_PL_,^53^*Φ*_PL_ –quantum yield of film, *η*_out_ – out-coupling efficiency (20%), *η*_rc_ – product of charge recombination efficiency (100%), *η*_γ_ – radiative exciton-production (25%)] and EUE can be estimated [*η*_s_ = *5* × *η*_ex_/*Φ*_PL_ × 100]: maximum exciton utilizing the efficiency of the devices based on CAPI and CCAPI have been calculated as 64 and 83% (Fig. 8 – CAPI and S_8_ – CCAPI†), respectively and exceed the 25% theoretical limit of spin statistics for conventional fluorescent OLEDs. The efficiency roll-off (*η*_roll-off_) is 0.92% only at a luminance of 1000 cd m^2^; however, the EQE is still not satisfactory for display applications. Therefore, the non-doped device with a configuration of ^b^ITO/PEDOT:PSS (40 nm)/TCTA (30 nm)/CAPI or CCAPI (20 nm)/TPBi(30 nm)/LiF (1 nm)/Al (100 nm) has been fabricated to enhance the efficiencies (Fig. S7†). The non-doped device with CAPI/CCAPI showed blue emission (467/450 nm) maximum current efficiency, power efficiency and EQE of 14.06/16.83; 14.81/15.32; 8.89/12.0, respectively, with a luminance of 28 801/32 546 cd m^−2^ and the efficiency roll-off (*η*_roll-off_) was 0.53% only at a luminance of 1000 cd m^−2^. The high EQE and low roll-off efficiency further emphasized the great potential of new CAPI and CCAPI materials for industrial applications. The EL spectra are stable with a driving voltage range of 3 V to 10 V (Fig. 7). The transient EL decay curves of devices with the configuration of ^b^CAPI/^b^CCAPI at different voltages correspond to two components: rapid EL decay originating from the fluorescence of S_1_ and delayed decay. The ratio of the delayed component decreased from 3 V to 10 V because of the quenching of dark triplet excitons and the amplified delayed component obtained from the plot of log EL intensity and log time [slope: −0.21 (initial time); −1.46 (log time)] (Fig. 3);^58^ however, the slope should be −2 instead of −1.46 for the TTA mechanism. Therefore, the TTA configuration is insignificant in the EL process. The TPAANPPI exhibited *η*_c_, *η*_p_ and EQE of 12.51 cd A^−1^, 11.47 lm W^−1^ and 8.00%, respectively.^51^

**Fig. 9:**
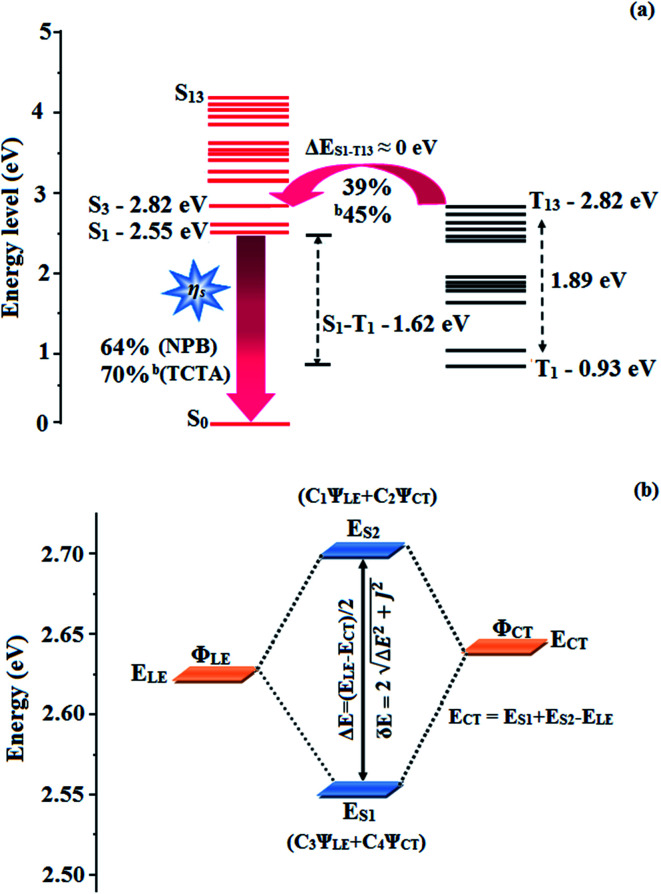
(a) Reverse intersystem crossing; (b) hybridization process of LE and CT states of CAPI.

The small energy splitting (Δ*E*_ST_ ≈ 0) between S_3_ and T_13_ (0.00 eV – CAPI) (Fig. 9) and S_4_ and T_8_ (0.00 eV – CCAPI) (Fig. S9†) promotes potential RISC processes and the dark triplet excitons are converted into singlet excitons in the EL process [^a^CAPI/^b^CAPI – 39/45%; ^a^CCAPI/^b^CCAPI – 58/70%]. According to the energy-gap law,^59^ the larger energy-gap between T_13_ and T_1_ of CAPI (1.89 eV) and T_8_ and T_1_ of CCAPI (1.08 eV) inhibits the internal conversion (IC) process and the more competitive RISC process between T_13_ and S_3_ of CAPI (0.00 eV) and T_8_ and S_4_ of CCAPI (0.00 eV) is predominant because of a narrower energy gap. Therefore, a large fraction of electro-generated dark triplet excitons were transferred into singlet excitons *via* T_13_ → S_3_ (CAPI) and T_8_ → S_4_ (CCAPI) channels. Moreover, the larger energy gap between S_1_ and T_1_ (Δ*E*_S_1_T_1__ = 1.62 eV – CAPI and 0.51 eV – CCAPI) prevents the intersystem crossing process from low-lying S_1_ to T_1_ and excludes the existence of the TADF mechanism. To the best of our knowledge, the efficiencies of CAPI and CCAPI are comparable with those of blue-emitting devices reported recently (Table 4).^1–4,60–75^ Therefore, the HLCT mechanism is likely responsible for the radiative exciton ratio above 25% in the non-doped OLED based on CAPI and CCAPI.

**Table 4:** Summary of device efficiencies

Emissive materials	*V* (V)	*η*_c_ (cd A^−1^)	*η*_p_ (lm W^−1^)	EL (nm)	EQE_max_ (%)	*L*_max_ (cd m^−2^)	CIE (*x*,*y*)	Ref.
CAPI^a^	3.00	13.32	13.0	467	8.4	26 490	0.15,0.20	This work
CAPI^b^	2.90	14.06	14.81	467	8.89	28 801	0.15,0.20	This work
CCAPI^a^	2.87	15.26	13.89	450	10.5	30 628	0.15,0.20	This work
CCAPI^b^	2.80	16.83	15.32	450	12.0	32 546	0.15,0.20	This work
PIAnCN	3.0	13.6	—	470	9.44	577 787	0.14,0.19	1
TPA-AN	—	5.06	2.48	460	3.0	10 079	0.15,0.23	2
2		3.26	2.92	454	2.05	1487	0.18,0.18	3
*m*-MethylTPE-pTPE	4.0	8.4	—	452	2.60	11 668	0.18,0.21	4
*o*-Methyl-BTPE	4.0	6.7	—	450	1.57	8685	0.18,0.18	4
*o*-MethylTPE-*p*TPE	3.5	9.7	—	454	4.06	14 644	0.17,0.21	4
	4.3	3.88	1.33	436/464/492	1.68	11 629	0.18,0.22	59
PhN-O	4.0	4.61	—	460	3.09	14 747	0.17,0.19	60
PhN	4.0	3.65	—	470	2.27	7707	0.17,0.23	60
1	—	0.93	1.01	460	—	1525	0.18,0.23	61
2	—	1.59	1.46	451	—	2011	0.17,0.21	61
3	—	1.12	0.93	445	—	1303	0.17,0.19	61
BDNPA	—	5.21	—	—	—	1300	0.16,0.19	62
Anthracene-PI	3.2	1.33	0.97	472	0.80	—	0.16,0.24	63
Py-BPI	2.5	3.27	3.17	468	2.07	—	0.15,0.18	63
Py-2NTF	3.9	2.50	1.37	456	1.37	6081	0.17,0.18	64
Chromophore I	7.0	0.28	—	460	—	279	0.19,0.22	65
Chromophore II	6.0	—	—	466	—	160	—	65
3	3.5	3.43	—	460	2.29	35 600	0.16,0.18	66
Py-TPICN	3.6	3.00	2.62	440	1.34	14 592	0.15,0.18	67
PPBC	—	1.29	1.34	441	—	7500	0.21,0.22	68
1	—	2.25	0.92	478	1.85	14 565	0.17,0.24	69
2	—	2.13	1.03	465	1.23	6163	0.16,0.23	69
DTPA-DSO2	4.5	9.1	5.4	—	6.3	—	0.14,0.22	70
DBPA-DSO_2_	3.0	6.5	6.8	—	4.7	—	0.17,0.22	70
6a	—	4.3	—	—	2.8	—	0.16,0.21	71
6b	—	4.2	—	—	2.6	—	0.16,0.22	71
6c	—	1.7	—	—	2.4	—	0.15,0.18	71
TSPI-1	3.77	—	—	457	—	1.6	0.17,0.18	72
BPPI	2.8	6.87	6.2	468	4.0	—	0.16,0.21	73
INaCPI	2.73	4.32	2.46	—	2.82	—	0.16,0.20	73
Py-BPI	2.5	3.27	3.17	—	2.07	—	0.15,0.18	73
PCz-an-PPI	2.6	6.43	6.23	—	3.99	—	0.15,0.23	73
DPF-TPI	2.7	8.41	7.23	—	4.85	—	0.17,0.24	73
CP-PPI	5.91	3.51	—	458	2.39	—	0.18,0.21	74

## 4 Conclusion

We have synthesized novel D–π–A materials with HLCT character enhancing the exciton utilization efficiency. The small energy splitting (Δ*E*_ST_ ≈ 0) between S_3_ and T_13_ (0.00 eV – CAPI) and S_4_ and T_8_ (0.00 eV – CCAPI) promotes a potential RISC process and the dark triplet excitons are effectively converted to singlet excitons in the EL process. The non-doped device with CCAPI shows blue emission (450 nm) with maximum current efficiency (*η*_c_), power efficiency (*η*_p_), external quantum efficiency (*η*_ex_) of 16.83 cd A^−1^, 15.32 lm W^−1^, 12.0%, respectively, and exciton utilization efficiency (EUE) of 95% with a luminance of 32 546 cd m^−2^ and a small roll-off efficiency of 0.53%. The amplified delayed component with obtained slope ruled out the contribution of the TTA mechanism and confirmed that the HLCT mechanism is responsible for the radiative exciton ratio above 25% in the non-doped OLED based on CAPI and CCAPI. The high EQE and small roll-off efficiency of devices emphasize the great potential of CAPI and CCAPI for industrial applications.

## Conflicts of interest

There are no conflicts to declare.

## Supplementary Material

RA-011-D0RA10934G-s001
