# Supplementary material for: Blue organic light-emitting diodes with hybridized local and charge-transfer excited state realizing high external quantum efficiency
Source: RSC Adv. 2021 Feb 24;11(15):8606–18. doi: 10.1039/d0ra10934g (PMC8695226; doi:10.1039/d0ra10934g)
Supplement: RA-011-D0RA10934G-s001 [file RA-011-D0RA10934G-s001.pdf]

Electronic Supplementary Material (ESI) for RSC Advances.
This journal is © The Royal Society of Chemistry 2021

# Supporting information

**Blue organic light emitting diodes with hybridised local and charge transfer excited state realizing high external quantum efficiency**

**Jayaraman Jayabharathi*, Shanmugam Thilagavathy , Venugopal Thanikachalam**

*Department of Chemistry, Annamalai University, Annamalai nagar, Tamilnadu- 608 002, India*

* Address for correspondence

Dr. J. Jayabharathi,FRSC
(UGC-BSR Mid – Career awardee)
Director, CIPR
Professor of Chemistry
Department of Chemistry
Annamalai University
Annamalai nagar 608 002
Tamilnadu, India.
Tel: +91 9443940735
E-mail: jtchalam2005@yahoo.co.in

# Contents

**SI- I: Measurements**

**SI-II: Device fabrication and measurement**

**SI–III: Computational details**

**SI–IV: Figures S1**

**SI–V: Charge-Transfer indexes (Figures S2-S9)**

**SI–VI: Tables (S1-S15)**

## SI- I: Measurements

$H^1$ and $C^{13}$ NMR measurements were recorded on Bruker 400 MHz spectrometer and mass spectra were recorded on Agilent LCMS VL SD. The UV-Vis spectra were recorded on Lambda 35 Perkin Elmer (solution)/ Lambda 35 spectrophotometer (RSA-PE-20) (film). The emission spectra have been recorded with Perkin Elmer LS55 spectrometer and quantum yield have been measured with fluorescence spectrometer (Model-F7100 with integrating sphere). The decomposition temperature ($T_d$) and $T_g$ (glass transition temperature) was measured with Perkin Elmer thermal analysis system (10° C $min^{-1}$; $N_2$ flow rate - 100 ml $min^{-1}$) and NETZSCH (DSC-204) (10° C $min^{-1}$; $N_2$ atmosphere), respectively. Fluorescence lifetime was estimated by time correlated single-photon counting (TCSPC) method on Horiba Fluorocube-01-NL lifetime system, nano LED is excitation source with TBX-PS is detector and DAS6 software has been used to analyze the decay by reconvolution method. Oxidation potential of emissive materials was measured from potentiostat electrochemical analyzer (Biologic science instrument SP 200). Ferrocene was used as internal standard with HOMO of -4.80 eV and 0.1M tetrabutylammoniumperchlorate in $CH_2Cl_2$ as supporting electrolyte.

## SI-II: Device fabrication and measurement

ITO glass (resistance 20 Ω/sq) were cleaned with acetone, deionized water and isopropanol, dried (120 $^oC$) followed by UV-zone treatment (20 min) and transferred into deposition system. The devices were fabricated by the multiple source beam deposition method in vacuum at a pressure of $4\times10^{-5}$ mbar. Evaporation rates of 2-4 Å $s^{-1}$ (organic materials) and 0.1 and 4 Å $s^{-1}$ for LiF and metal electrodes were applied, respectively. The thickness of each deposition layer was monitored with quartz crystal thickness monitor. The EL measurement with CIE coordinates was recorded with USB-650-VIS-NIR spectrometer (Ocean Optics, Inc, USA). The current density-voltage-luminance (J-V-L) characteristics was performed using source meter (Keithley 2450) equipped with LS-110 light intensity meter. The

external quantum efficiency was determined from luminance, current density and EL spectrum assuming Lambertian distribution.

## SI–III: Computational details

The ground ($S_0$) (DFT) / excited ($S_n$*) (TD-DFT) states of emissive materials were analyzed by Gaussian 09 program [40] and multifunctional wavefunction analyzer (Multiwfn) [40]. The natural transition orbitals (HONTOs & LUNTOs) with hole-particle contribution were studied in detail. The TDM (transition density matrix) map of emissive materials was analyzed to support HLCT character.

**Scheme S1.** Synthetic route of CAPI and CCAPI.

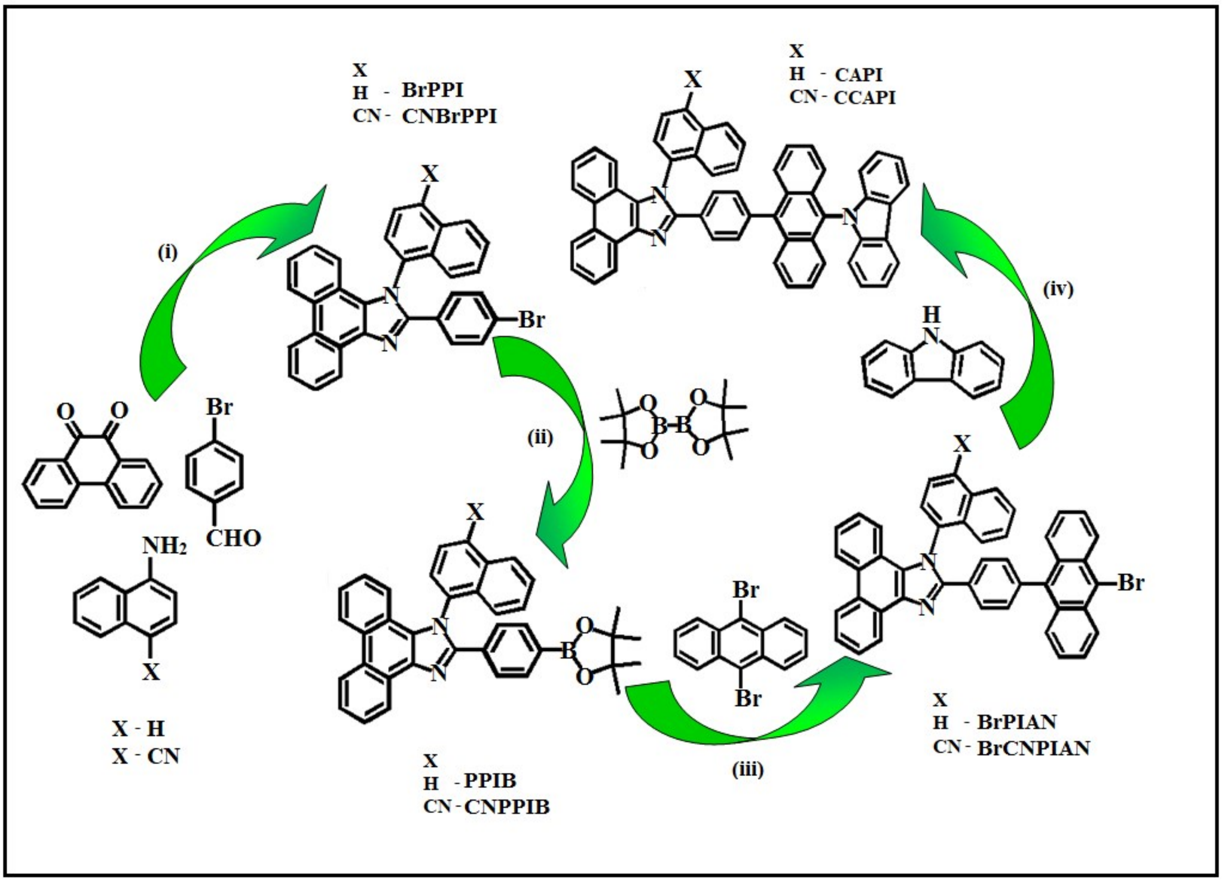


(i) $CH_3COONH_4$, $CH_3COOH$, 120°C, $N_2$, 12h; (ii) Pd(dppf)$Cl_2$, KOAc, 1,4-dioxane, 90°C, $N_2$, 48h;
(iii) $K_2CO_3$, $Pd(PPh_3)_4$, THF, $H_2O$, 48h, 70°C, $N_2$; (iv)$K_2CO_3$, $Pd(PPh_3)_4$, THF, $H_2O$, 48h, 70°C, $N_2$

## SI–IV: Figures

**Figure S1.** (a) Solvatochromism: Absorption and (b) Emission spectra

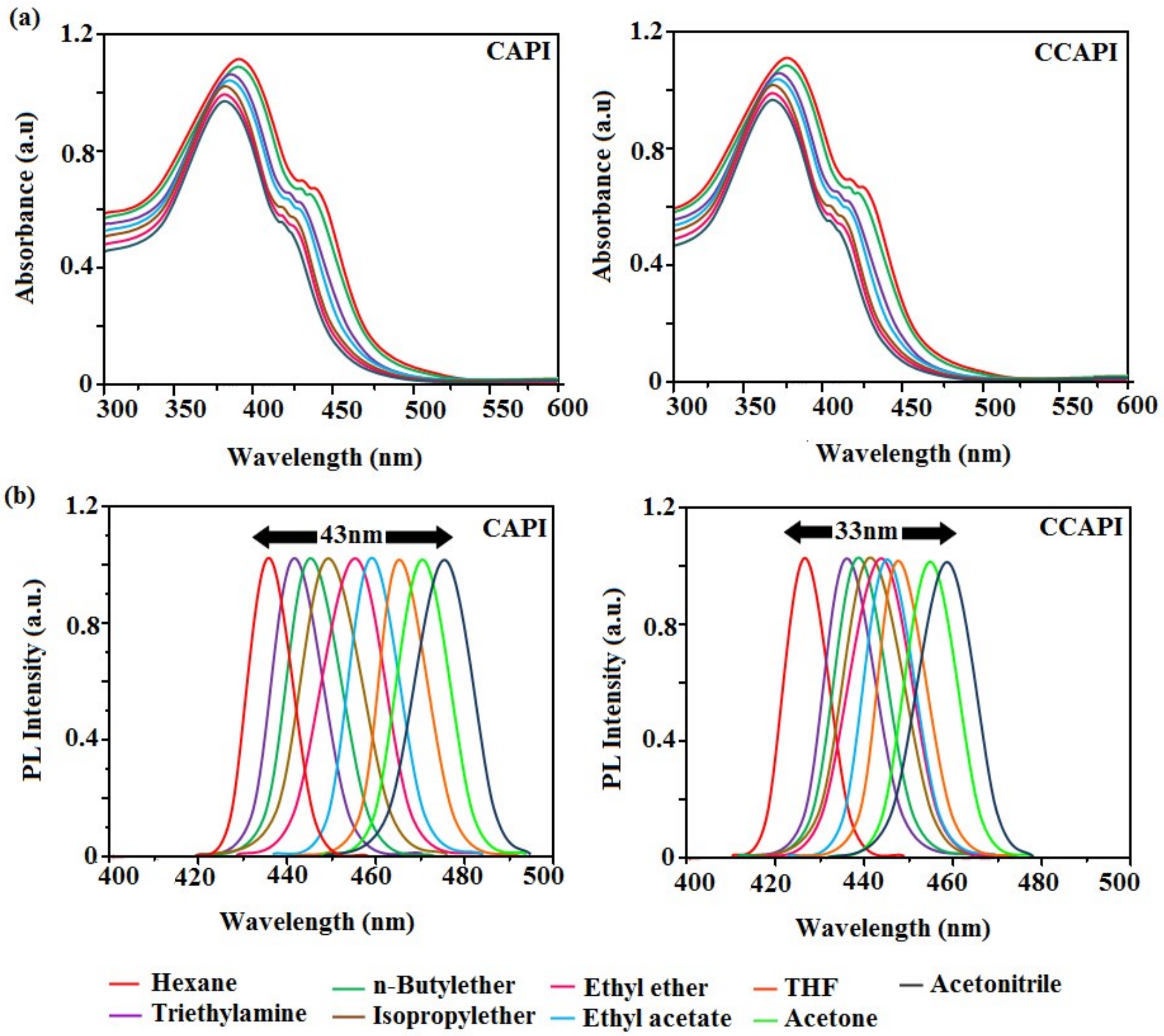

**SI–V: Charge-Transfer intexes**

The hole-particle pair interactions have been related to the distance covered during the excitations one possible descriptor Δr intex could be used to calculate the average distance which is weighted in function of the excitation coefficients.

$$\Delta r = \frac{\Sigma_{ia} k_{ia}^2 \left| \langle \varphi_a | r | \varphi_a \rangle - \langle \varphi_i | r | \varphi_i \rangle \right|}{\Sigma_{ia} K_{ia}^2}$$ …………….. (S1)

Where $\left| \langle \varphi_i | r | \varphi_i \rangle \right|$ is the norm of the orbital centroid [1–4]. Δr–index will be expressed in Å. The density variation associated to the electronic transition is given by

$$\Delta\rho(r) = \rho_{EX}(r) - \rho_{GS}(r)$$ ..................... (S2)

Where $\rho_{GS}(r)$ and $\rho_{EX}(r)$ are the electron densities of to the ground and excited states, respectively. Two functions, $\rho_+(r)$ and $\rho_-(r)$, corresponds to the points in space where an increment or a depletion of the density upon absorption is produced and they can be defined as follows:

$$\rho_+(r) = \begin{cases} \Delta\rho(r) & if\ \Delta\rho(r) > 0 \\ 0 & if\ \Delta\rho(r) < 0 \end{cases}$$ ........................ (S3)

$$\rho_-(r) = \begin{cases} \Delta\rho(r) & if\ \Delta\rho(r) < 0 \\ 0 & if\ \Delta\rho(r) > 0 \end{cases}$$ ......................... (S4)

The barycenters of the spatial regions $R_+$ and $R_-$ are related with $\rho_+(r)$ and $\rho_-(r)$ and are shown as

$$R_+ = \frac{\int r\rho_+(r)dr}{\int \rho_+(r)dr} = (x_+, y_+, z_+)$$ .................... (S5)

$$R_- = \frac{\int r\rho_-(r)dr}{\int \rho_-(r)dr} = (x_-, y_-, z_-)$$ ...................... (S6)

The spatial distance ($D_{CT}$) between the two barycenters $R_+$ and $R_–$ of density distributions can thus be used to measure the CT excitation length

$$D_{CT} = |R_+ - R_-| \quad \text{(S7)}$$

The transferred charge ($q_{CT}$) can be obtained by integrating over all space $\rho_+ (\rho_-)$. Variation in dipole moment between the ground and the excited states ($\mu_{CT}$) can be computed by the following relation:

$$\|\mu_{CT}\| = D_{CT}\int \rho_+(r)dr = D_{CT}\int \rho_-(r)dr \quad \text{(S8)}$$

$$= D_{CT}q_{CT} \quad \text{(S9)}$$

The difference between the dipole moments $\|\mu_{CT}\|$ have been computed for the ground and the excited states $\Delta\mu_{ES–GS}$. The two centroids of charges ($C^+/C^–$) associated to the positive and negative density regions are calculated as follows. First the root-mean-square deviations along the three axis ($\sigma_{aj}$, j = x, y, z; a = + or –) are computed as

$$\sigma_{a,j} = \sqrt{\frac{\sum_i \rho_a(r_i)(j_i - j_a)^2}{\sum_i \rho_a(r_i)}} \quad \text{(S10)}$$

The two centroids ($C_+$ and $C_–$) are defined as

$$C_+(r) = A_+ e\left(-\frac{(x - x_+)^2}{2\sigma_{+x}^2} - \frac{(y - y_+)^2}{2\sigma_{+y}^2} - \frac{(z - z_+)^2}{2\sigma_{+z}^2}\right) \quad \text{(S11)}$$

$$C_{-+}(r) = A_- e\left(-\frac{(x - x_-)^2}{2\sigma_{-x}^2} - \frac{(y - y_-)^2}{2\sigma_{-y}^2} - \frac{(z - z_-)^2}{2\sigma_{-z}^2}\right) \quad \text{(S12)}$$

The normalization factors (A+ and A_) are used to impose the integrated charge on the centroid to be equal to the corresponding density change integrated in the whole space:

$$A_{+} = \frac{\int \rho_{+}(r)dr}{\int e(-\frac{(x - x_{-})^2}{2\sigma_{+x}^2} - \frac{(y - y_{-})^2}{2\sigma_{+y}^2} - \frac{(z - z_{-})^2}{2\sigma_{+z}^2})dr} \quad \text{.................. (S13)}$$

$$A_{-} = \frac{\int \rho_{-}(r)dr}{\int e(-\frac{(x - x_{-})^2}{2\sigma_{-x}^2} - \frac{(y - y_{-})^2}{2\sigma_{-y}^2} - \frac{(z - z_{-})^2}{2\sigma_{-z}^2})dr} \quad \text{.................. (S14)}$$

H index is defined as half of the sum of the centroids axis along the D–A direction, if the D–A direction is along the X axis, H is defined by the relation:

$$H = \frac{\sigma_{+x} + \sigma_{-x}}{2} \quad \text{.................... (S15)}$$

The centroid along X axis is expected. The t intex represents the difference between $D_{CT}$ and H:

$$t = D_{CT} - H \quad \text{............... (S16)}$$

**Figure S2.** Natural transition orbital pairs (HONTOs and LUNTOs) with transition character analysis for singlet and triplet states of CAPI.

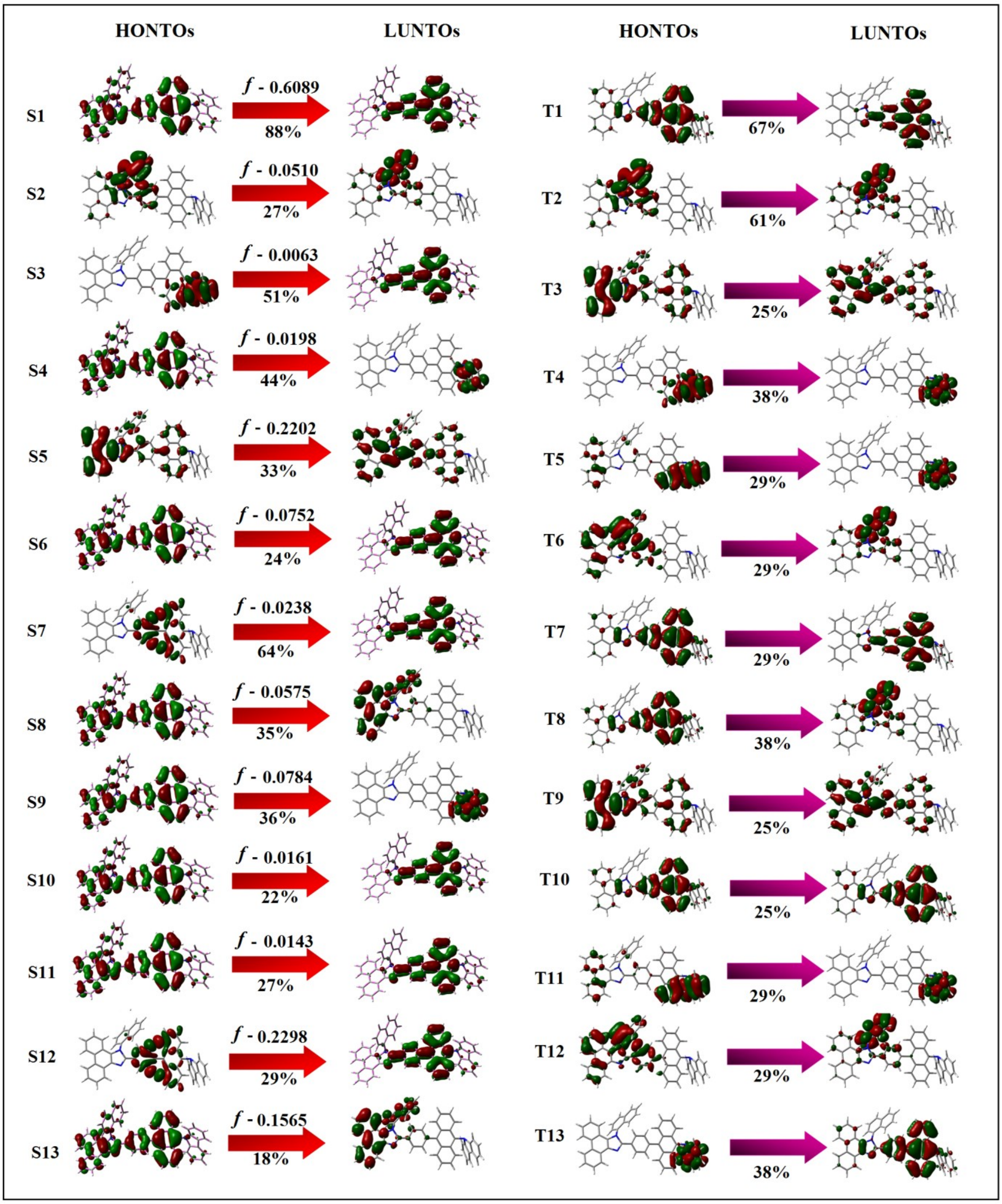
HONTOs
LUNTOs
HONTOs
LUNTOs
S1
f - 0.6089
88%
T1
67%
S2
f - 0.0510
27%
T2
61%
S3
f - 0.0063
51%
T3
25%
S4
f - 0.0198
44%
T4
38%
S5
f - 0.2202
33%
T5
29%
S6
f - 0.0752
24%
T6
29%
S7
f - 0.0238
64%
T7
29%
S8
f - 0.0575
35%
T8
38%
S9
f - 0.0784
36%
T9
25%
S10
f - 0.0161
22%
T10
25%
S11
f - 0.0143
27%
T11
29%
S12
f - 0.2298
29%
T12
29%
S13
f - 0.1565
18%
T13
38%

**Figure S3.** Natural transition orbital pairs (HONTOs and LUNTOs) with transition character analysis for singlet and triplet states of CCAPI.

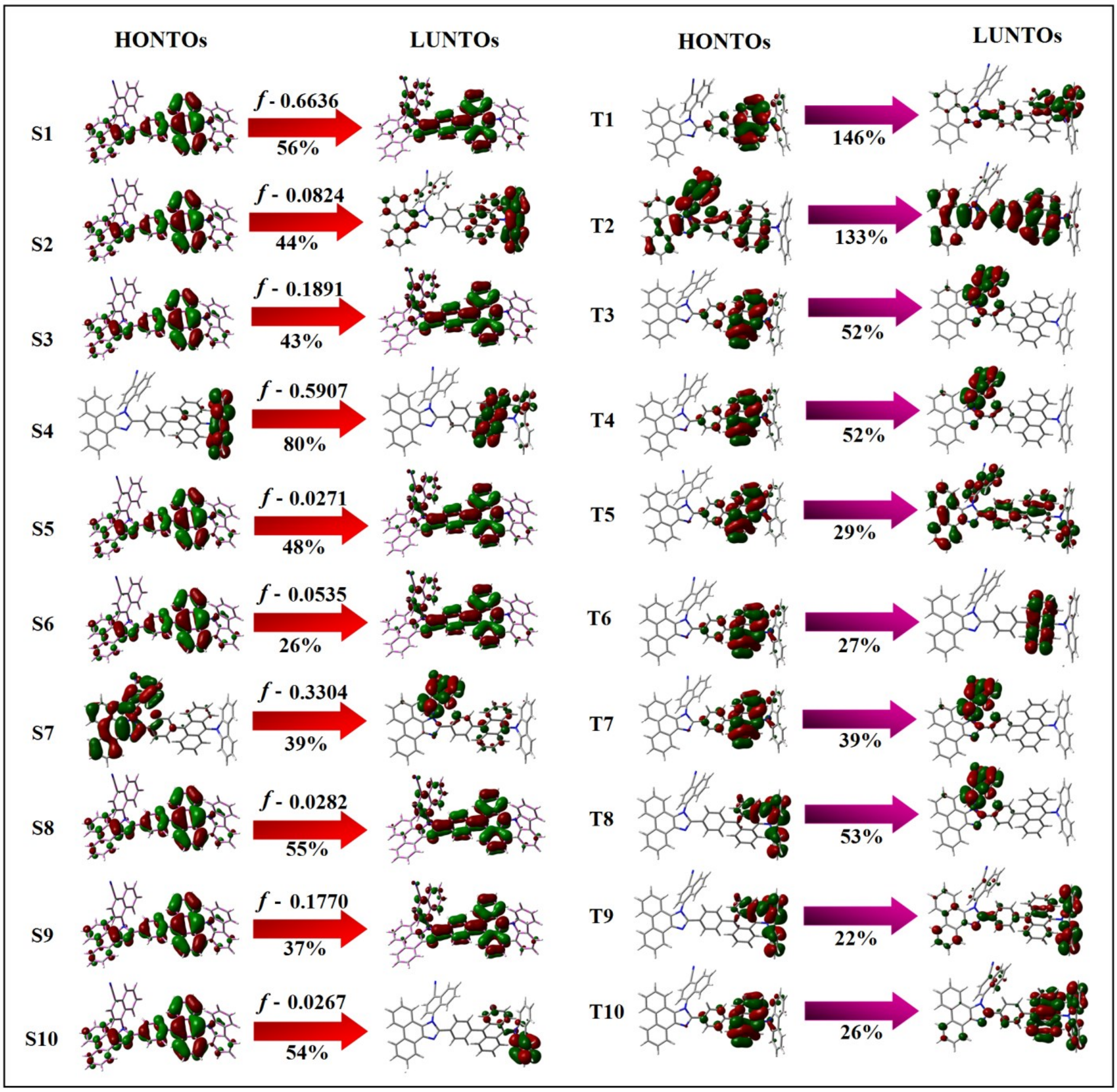

**Figure S4.** Hole and particle distribution and Transition density matrices (TDM) of CAPI.

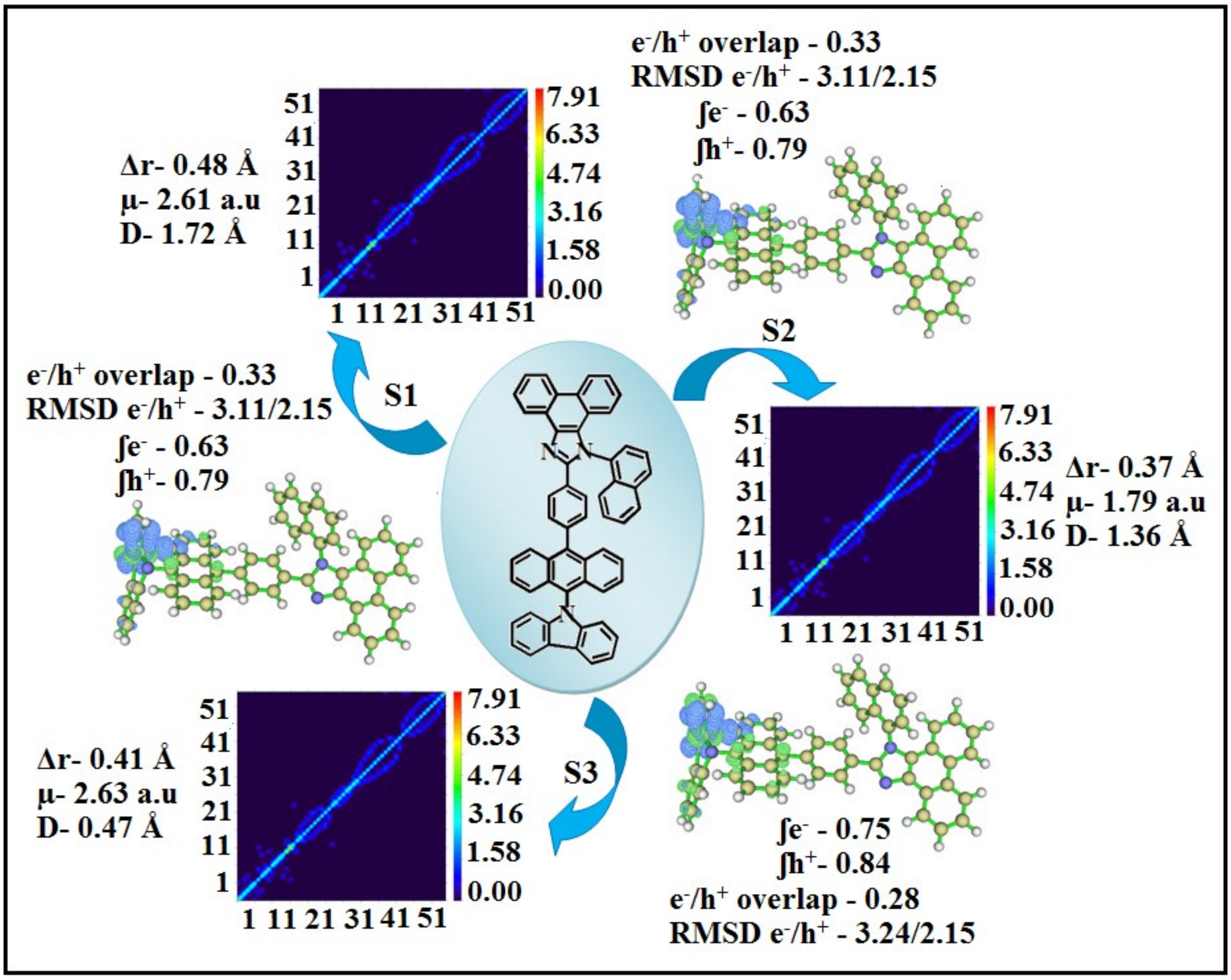

**Figure S5.** Hole and particle distribution and Transition density matrices (TDM) of CCAPI.

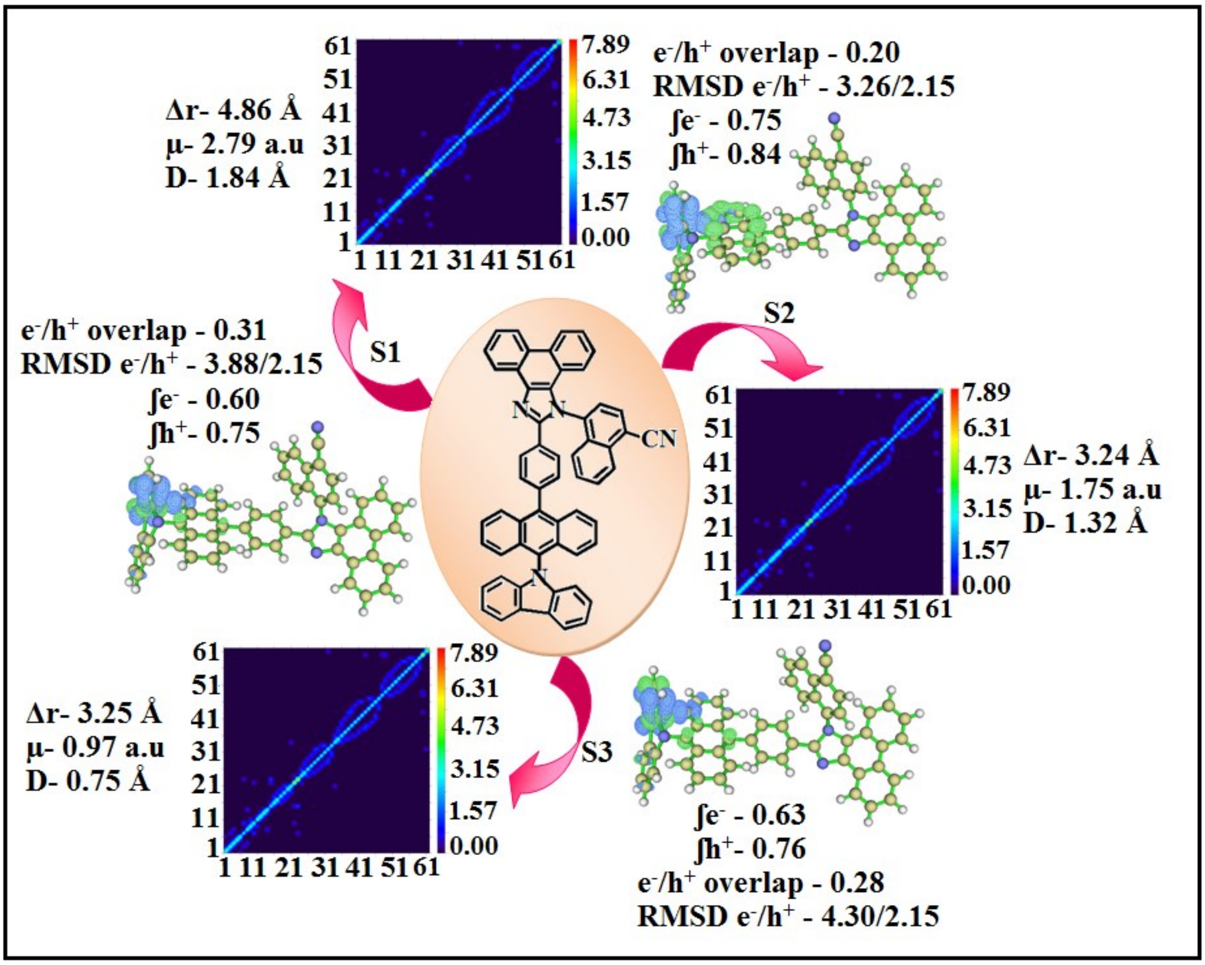

**Figure S6.** Potential energy surface scan (PES) diagram of (a) CAPI and CCAPI; (b) PES diagram of CAPI with increasing solvent polarity; (c) PES diagram of CCAPI with increasing solvent polarity.

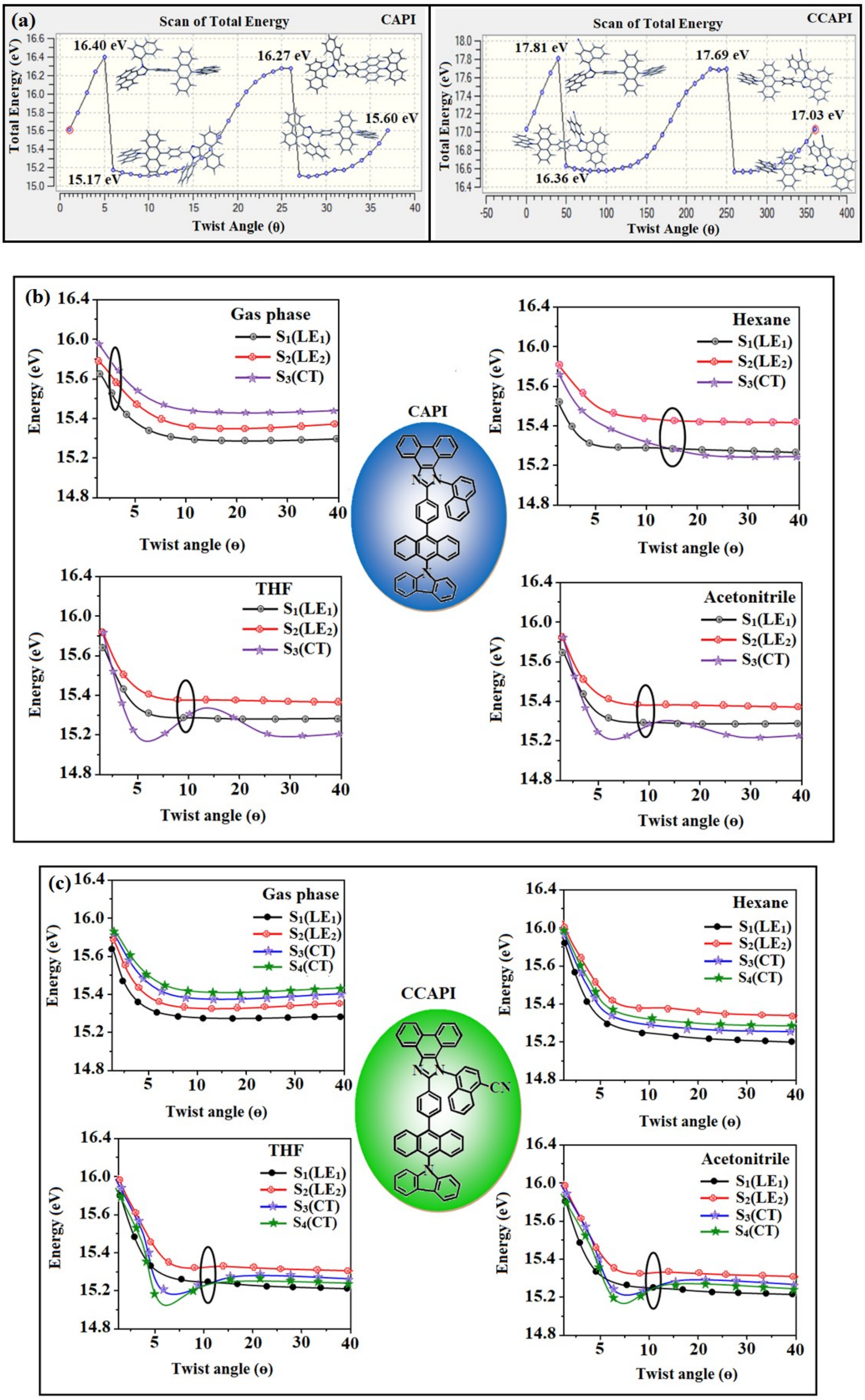

**Figure S7.** (a) Energy level diagram of non-doped device and (b) Configuration of non-doped device.

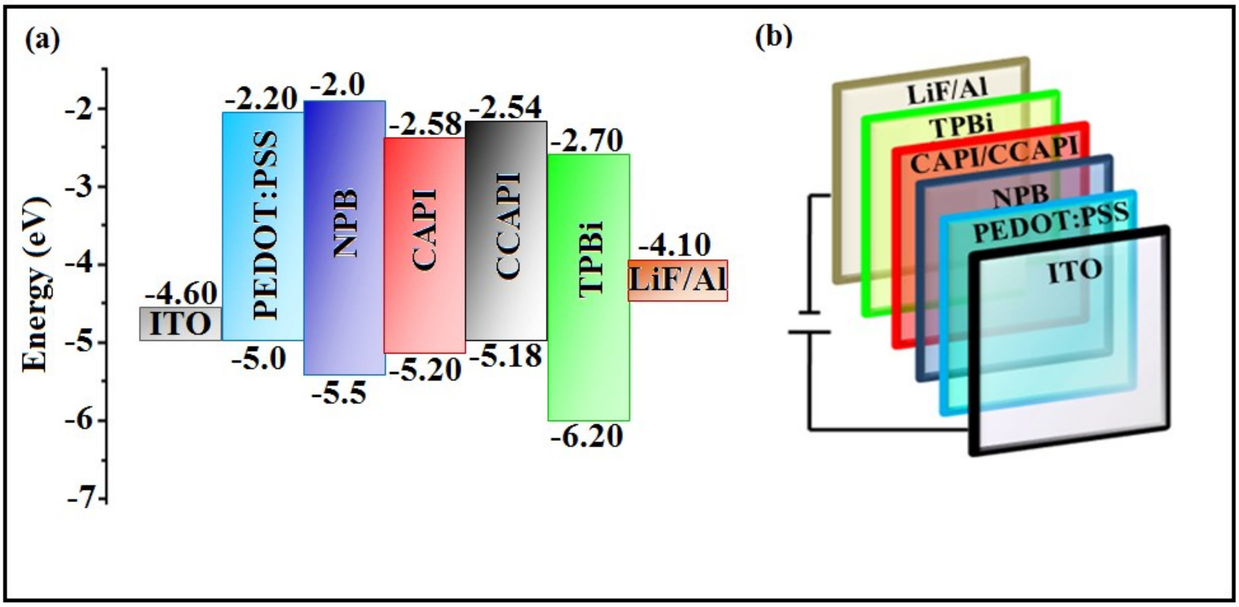

**Figure S8.** Scheme of hole and electron recombination in OLEDs of CCAPI.

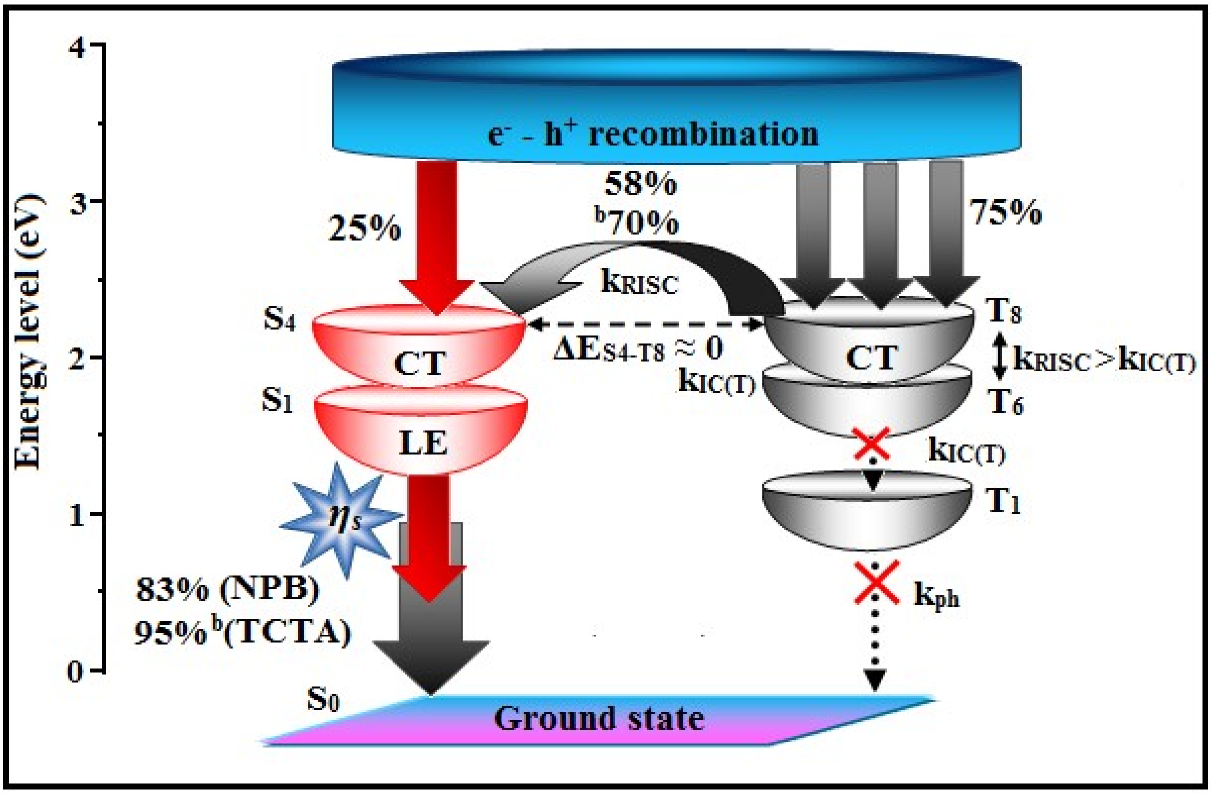

**Figure S9.** (a) Reverse intersystem crossing; (b) Hybridization process of LE and CT states of CCAPI.

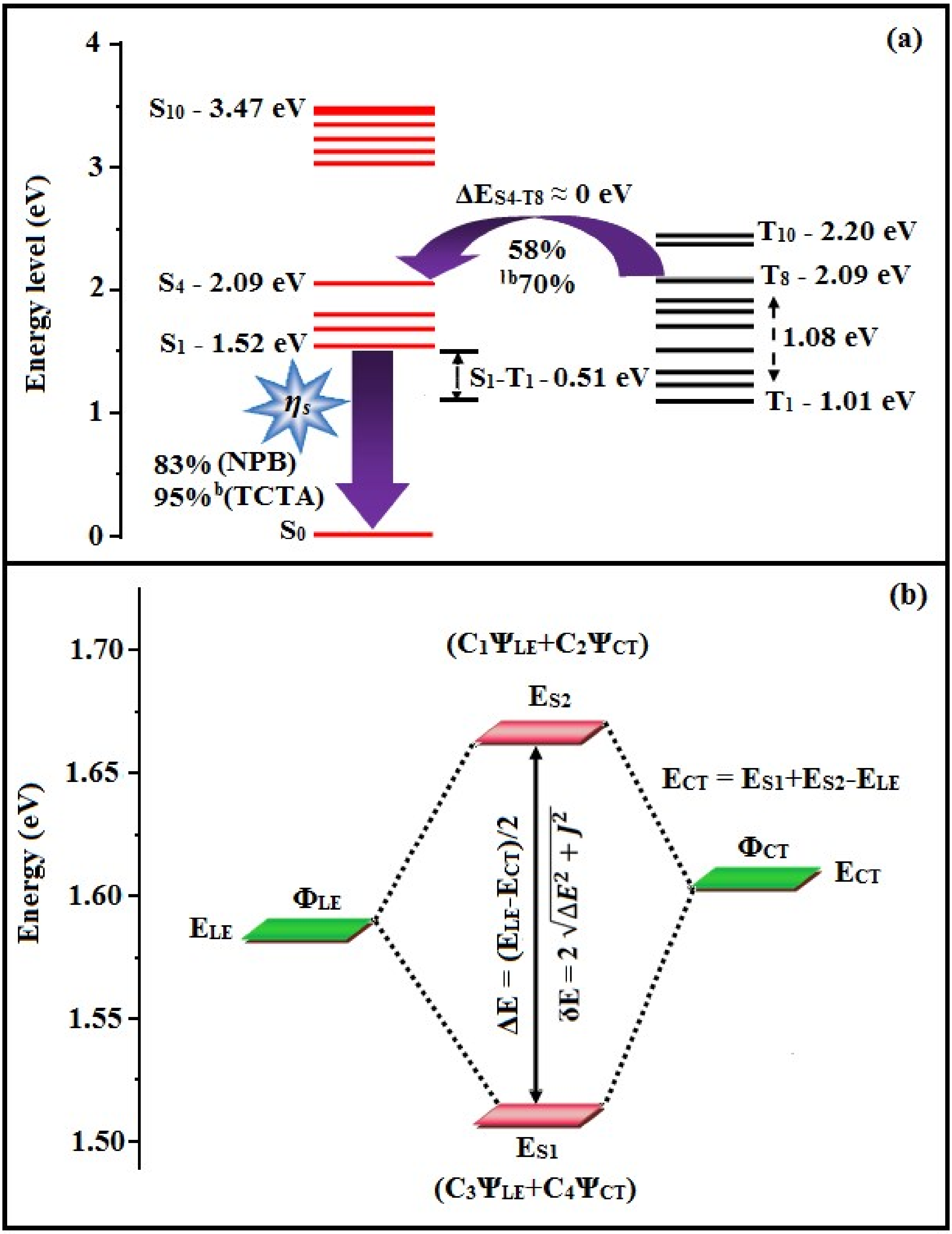

## SI–VI: Tables

**Table S1:** Photophysical properties of CAPI in different solvents.

| Solvents | ε | n | f (ε,n) | $\lambda_{ab}$ (nm) | $\upsilon_{ab}$ ($cm^{-1}$) | $\lambda_{flu}$ (nm) | $\upsilon_{flu}$ ($cm^{-1}$) | $\upsilon_{ss}$ ($cm^{-1}$) |
|---|---|---|---|---|---|---|---|---|
| Hexane | 2.42 | 1.40 | 0.0004 | 385 | 25974.03 | 435 | 22988.51 | 3085.52 |
| Triethylamine | 3.39 | 1.47 | 0.048 | 386 | 25906.74 | 445 | 22471.91 | 3434.826 |
| n-butyl ether | 3.08 | 1.39 | 0.096 | 387 | 25839.79 | 448 | 22321.43 | 3518.365 |
| Isopropyl ether | 4.33 | 1.35 | 0.145 | 385 | 25974.03 | 450 | 22222.22 | 3751.804 |
| Ethyl ether | 6.09 | 1.41 | 0.167 | 385 | 30395.14 | 458 | 23978.02 | 3459.801 |
| ethyl acetate | 7.52 | 1.40 | 0.186 | 386 | 25906.74 | 460 | 21739.13 | 4167.605 |
| THF | 9.08 | 1.42 | 0.209 | 387 | 25839.79 | 466 | 21459.23 | 4380.566 |
| Acetone | 36.7 | 1.42 | 0.285 | 386 | 25906.74 | 474 | 21097.05 | 4809.689 |
| acetonitrile | 37.5 | 1.34 | 0.305 | 385 | 25974.03 | 478 | 20920.5 | 5053.524 |

**Table S2:** Photophysical properties of CCAPI in different solvents.

| Solvents | ε | n | f (ε,n) | $\lambda_{ab}$ (nm) | $\upsilon_{ab}$ ($cm^{-1}$) | $\lambda_{flu}$ (nm) | $\upsilon_{flu}$ ($cm^{-1}$) | $\upsilon_{ss}$ ($cm^{-1}$) |
|---|---|---|---|---|---|---|---|---|
| Hexane | 2.42 | 1.40 | 0.0004 | 378 | 26455.03 | 427 | 23419.2 | 3135.823 |
| Triethylamine | 3.39 | 1.47 | 0.048 | 379 | 26385.22 | 438 | 22831.05 | 3554.174 |
| n-butyl ether | 3.08 | 1.39 | 0.096 | 380 | 26315.79 | 440 | 22727.27 | 3588.517 |
| Isopropyl ether | 4.33 | 1.35 | 0.145 | 378 | 26455.03 | 443 | 22573.36 | 3881.663 |
| Ethyl ether | 6.09 | 1.41 | 0.167 | 378 | 30395.14 | 447 | 22978.02 | 3481.101 |
| ethyl acetate | 7.52 | 1.40 | 0.186 | 379 | 26385.22 | 449 | 22271.71 | 4113.509 |
| THF | 9.08 | 1.42 | 0.209 | 380 | 26315.79 | 450 | 22222.22 | 4093.567 |
| Acetone | 36.7 | 1.42 | 0.285 | 379 | 26385.22 | 458 | 21834.06 | 4551.163 |
| acetonitrile | 37.5 | 1.34 | 0.305 | 378 | 26455.03 | 460 | 21739.13 | 4715.896 |

**Table S3.** Computed [zindo (Singlet or Triplet, n states=10)] singlet ($E_S$) and triplet ($E_T$) energies (eV), oscillator strength (*f*), dipole moment (μ, D) and singlet-triplet splitting ($\Delta E_{ST}$, eV) of CAPI from NTOs.

| Energy level | $E_S$ | Oscillator strength (f) | μ | NTO Transitions | $E_T$ | $\Delta E_{ST}$ | NTO Transitions |
|---|---|---|---|---|---|---|---|
| 1 | 2.55 | 0.7440 | 0.24 | 88% 139 → 140 | 0.93 | 1.62 | 67% 139 → 140 |
| 2 | 2.69 | 0.0510 | 0.51 | 27% 137 → 141 | 1.15 | 1.14 | 61% 137 → 141 |
| 3 | 2.82 | 0.0063 | 0.57 | 51% 136 → 140 | 1.67 | 1.15 | 25% 138 → 142 |
| 4 | 3.19 | 0.0198 | 0.29 | 44% 139 → 147 | 1.81 | 1.38 | 38% 136 → 144 |
| 5 | 3.35 | 0.2202 | 0.20 | 33% 138 → 142 | 1.84 | 1.51 | 29% 135 → 144 |
| 6 | 3.43 | 0.0752 | 0.30 | 24% 137 → 141 | 1.88 | 1.55 | 29% 133 → 141 |
| 7 | 3.51 | 0.0238 | 0.56 | 64% 132 → 140 | 1.97 | 1.54 | 67% 139 → 140 |
| 8 | 3.60 | 0.0575 | 0.98 | 35% 138 → 143 | 2.13 | 0.04 | 61% 137 → 141 |
| 9 | 3.74 | 0.0784 | 0.42 | 36% 136 → 144 | 2.42 | 0.32 | 25% 138 → 142 |
| 10 | 3.80 | 0.0161 | 0.35 | 22% 137 → 140 | 2.48 | 1.32 | 25% 138 → 142 |
| 11 | 3.85 | 0.0143 | 1.55 | 27% 129 → 140 | 2.61 | 1.24 | 29% 139 → 140 |
| 12 | 3.90 | 0.2298 | 1.84 | 29% 135 → 140 | 2.73 | 1.17 | 29% 134 → 141 |
| 13 | 3.93 | 0.1565 | 1.88 | 18% 137 → 142 | 2.82 | 1.11 | 38% 136 → 144 |

**Table S4.** Computed [zindo (Singlet or Triplet, n states=10)] singlet ($E_S$) and triplet ($E_T$) energies (eV), oscillator strength (*f*), dipole moment (μ, D) and singlet-triplet splitting ($\Delta E_{ST}$, eV) of CCAPI from NTOs.

| Energy level | $E_S$ | Oscillator strength (f) | μ | NTO Transitions | $E_T$ | $\Delta E_{ST}$ | NTO Transitions |
|---|---|---|---|---|---|---|---|
| 1 | 1.52 | 0.0271 | 0.32 | 56% 143 → 144 | 1.01 | 0.51 | 146% 144 → 146 |
| 2 | 1.67 | 0.0824 | 0.83 | 44% 143 → 147 | 1.21 | 0.46 | 133% 142 → 143 |
| 3 | 1.98 | 0.1891 | 0.93 | 43% 143 → 153 | 1.23 | 0.75 | 52% 142 → 145 |
| 4 | 2.09 | 0.5907 | 3.39 | 80% 142 → 144 | 1.52 | 0.57 | 52% 144 → 154 |
| 5 | 3.00 | 0.0271 | 0.40 | 48% 143 → 151 | 1.69 | 1.31 | 29% 144 → 152 |
| 6 | 3.12 | 0.0535 | 3.14 | 26% 143 → 151 | 1.83 | 1.29 | 27% 144 → 158 |
| 7 | 3.24 | 0.3304 | 0.56 | 39% 141 → 145 | 1.97 | 1.27 | 39% 144 → 158 |
| 8 | 3.36 | 0.0282 | 0.56 | 55% 142 → 151 | 2.09 | 1.27 | 53% 144 → 158 |
| 9 | 3.46 | 0.1770 | 0.95 | 22% 143 → 156 | 2.16 | 1.30 | 22% 140 → 153 |
| 10 | 3.47 | 0.0269 | 0.81 | 54% 143 → 154 | 2.20 | 1.27 | 26% 144 → 148 |

The integral value of hole and electron of CAPI (Table S5- singlet; TableS6-triplet) is higher than CCAPI (Table S7- singlet; TableS8-triplet) with transition density. The integral overlap of hole-electron distribution (S) is a measure of spatial separation of hole and electron. The integral overlap (S) of hole and electron and distance (D) between centroids of hole and electron evidences the existence of LE and CT states. The emitters CAPI and CCAPI show small D and high S, however, high D and high S of CAPI on comparison with CCAPI indicates higher charge transfer (CT) for CAPI . The variation of dipolemoment with respect to $S_0$ state is outputted which is directly evaluated based on the position of centroid of hole and electron. RMSD of hole or electron characterizes their distribution breadth: RMSD of both electron and hole in CAPI (Tables S9 & S10) and CCAPI(Tables S11 & S12) is higher in Y direction indicates electron and hole distribution is much broader in Y direction whereas RMSD of electron and hole of CCAPI is higher than CAPI. The overlap between the region of density depletion and increment have been visualised by using two centroids of charges (equations S9 and S10). Using this arbitrarily condensed function, computed overlap of $\rho+$ and $\rho-$ regions for CAPI and CCAPI is 0.9980 and 0.9962, respectively (Table S15) and H index of CAPI is higher than CCAPI. The CT intex, *i.e., t* index difference between $D_{CT}$ and H index is another measure of the separation of hole-electron (equations); *t* index of CCAPI is higher than CAPI. The $D_{CT}/\mu_{CT}$ of CAPI and CCAPI was calculated to be 0.332/ 37.28 and 0.133/15.07, respectively (Figure 4: Table S15). For both DDPPPA and DDPBA, the non-zero *t* is negative in all directions: overlap of hole and electron is very severe and eign value is greater than 0.96, supports the hybridization with dominant excitation pair in term of 94% of transition.

**Table S5:** Computed hole and electron overlap (S), distance between centroids of hole and electron (D, Å) and dipole moment (μ) for singlet states of CAPI.

| State | Hole integral | Electron integral | Integral of transition density | Integral overlap of $h^+$- $e^-$ (S) | Centroid of hole (Å) | | | Centroid of electron (Å) | | | D (Å) | μ (a.u) |
|---|---|---|---|---|---|---|---|---|---|---|---|---|
| | | | | | x | y | z | x | y | z | | |
| S1 | 0.7582 | 0.5047 | -0.0095 | 0.1775 | -2.0284 | -3.8305 | 2.1826 | -1.7175 | -4.1161 | 2.2644 | 0.43 | 0.51 |
| S2 | 0.8782 | 0.7273 | 0.0018 | 0.5822 | 4.6385 | 3.5532 | 0.0529 | 5.2460 | 3.6272 | -0.0100 | 0.61 | 0.93 |
| S3 | 0.8549 | 0.6978 | 0.0003 | 0.5129 | 3.4878 | -0.4737 | 0.0012 | 3.3615 | -0.8073 | -0.0967 | 0.36 | 0.54 |
| S4 | 0.7608 | 0.5662 | 0.0016 | 0.4827 | 4.5179 | 4.4771 | 0.0570 | 5.3327 | 5.5388 | 0.0093 | 1.33 | 1.67 |
| S5 | 0.7420 | 0.5856 | 0.0035 | 0.3399 | 1.9813 | -1.5988 | 0.0592 | -0.0506 | -1.8080 | -0.0315 | 2.04 | 2.56 |
| S6 | 0.7041 | 0.5178 | 0.0010 | 0.4434 | 4.1592 | -4.4182 | -0.2014 | 3.6564 | -4.1301 | -0.2078 | 0.57 | 0.66 |
| S7 | 0.5742 | 0.4369 | 0.0035 | 0.2966 | 2.4688 | -0.5593 | 0.1268 | 2.3253 | -0.7205 | 0.0813 | 0.22 | 0.21 |
| S8 | 0.7232 | 0.6790 | -0.0027 | 0.1395 | 2.6389 | 4.5505 | 0.3070 | 4.9704 | 6.0034 | 0.1086 | 2.75 | 3.64 |
| S9 | 0.7017 | 0.5324 | -0.0054 | 0.3412 | -2.0744 | 0.7939 | -0.2136 | -1.1353 | 0.4840 | 0.0674 | 1.02 | 1.19 |
| S10 | 0.5686 | 0.4170 | -0.0038 | 0.2914 | -1.6902 | 0.8456 | -0.0593 | -2.2545 | 0.4756 | -0.0592 | 0.67 | 0.62 |
| S11 | 0.6162 | 0.5611 | 0.0029 | 0.1511 | -0.7873 | 0.1245 | -0.2029 | -2.1797 | -0.0262 | -0.1573 | 1.40 | 1.55 |
| S12 | 0.7011 | 0.5309 | -0.0064 | 0.3232 | 2.8224 | 0.5347 | -0.1201 | 2.5689 | 2.0947 | -0.0966 | 1.58 | 1.84 |
| S13 | 0.7721 | 0.5772 | 0.0061 | 0.4074 | 1.1144 | -0.4059 | -0.1082 | 2.5921 | -0.4941 | -0.1497 | 1.48 | 1.88 |

**Table S6:** Computed hole and electron overlap (S), distance between centroids of hole and electron (D, Å) and dipole moment (μ) for triplet states of CAPI.

| State | Hole integral | Electron integral | Integral of transition density | Integral overlap of $h^+$- $e^-$ (S) | Centroid of hole (Å) | | | Centroid of electron (Å) | | | D (Å) | μ (a.u) |
|---|---|---|---|---|---|---|---|---|---|---|---|---|
| | | | | | x | y | z | x | y | z | | |
| T1 | 0.7593 | 0.6111 | 0.0012 | 0.5229 | 5.3239 | 5.7740 | 0.0469 | 5.5117 | 5.7606 | 0.0368 | 0.19 | 0.24 |
| T2 | 0.6974 | 0.5586 | 0.0033 | 0.4637 | 4.6972 | -5.7342 | -0.2867 | 4.4411 | -5.3899 | -0.2474 | 0.43 | 0.51 |
| T3 | 0.4361 | 0.3365 | 0.0020 | 0.2191 | -1.3452 | 0.6366 | 0.0619 | -2.1211 | 0.6907 | 0.0845 | 0.78 | 0.57 |
| T4 | 0.2418 | 0.1731 | -0.0043 | 0.1276 | -3.6779 | 0.4487 | -0.3056 | -3.0428 | 0.0793 | -0.1777 | 0.74 | 0.29 |
| T5 | 0.2237 | 0.1559 | -0.0052 | 0.1151 | -3.4828 | -0.2566 | -0.3592 | -3.8496 | 0.1283 | -0.2165 | 0.55 | 0.20 |
| T6 | 0.6066 | 0.4239 | 0.0055 | 0.3667 | 4.4420 | -5.2823 | -0.2400 | 4.3843 | -4.9832 | -0.2104 | 0.31 | 0.30 |
| T7 | 0.4380 | 0.2813 | 0.0007 | 0.2510 | -6.3588 | 1.3311 | -0.1735 | -7.1210 | 1.0120 | -0.1458 | 0.83 | 0.56 |
| T8 | 0.4706 | 0.2985 | -0.0027 | 0.2655 | -7.0105 | 0.7439 | -0.0160 | -8.3512 | 0.6881 | 0.0629 | 1.34 | 0.98 |
| T9 | 0.7312 | 0.4885 | 0.0094 | 0.1725 | -2.0499 | -3.8592 | 2.1945 | -1.7186 | -4.0136 | 2.2043 | 0.36 | 0.42 |
| T10 | 0.7798 | 0.5377 | -0.0001 | 0.4836 | 5.2364 | 5.9467 | 0.0162 | 5.5042 | 5.8485 | 0.0128 | 0.28 | 0.35 |
| T11 | 0.4531 | 0.2811 | -0.0012 | 0.2474 | -2.4156 | -0.1142 | -0.0569 | -3.2210 | 0.0818 | -0.089 | 0.82 | 0.57 |
| T12 | 0.7127 | 0.4894 | -0.0089 | 0.3176 | 2.9208 | 2.3538 | -0.3508 | 2.8715 | 2.8739 | -0.3551 | 0.52 | 0.59 |
| T13 | 0.5150 | 0.3524 | 0.0046 | 0.2699 | 5.8053 | -1.2638 | -0.0499 | 6.0000 | -0.9504 | -0.0713 | 0.36 | 0.30 |

**Table S7:** Computed hole and electron overlap (S), distance between centroids of hole and electron (D, Å) and dipole moment (μ) for singlet states of CCAPI.

| State | Hole integral | Electron integral | Integral of transition density | Integral overlap of $h^+$- $e^-$ (S) | Centroid of hole (Å) | | | Centroid of electron (Å) | | | D (Å) | μ (a.u) |
|---|---|---|---|---|---|---|---|---|---|---|---|---|
| | | | | | x | y | z | x | y | z | | |
| S1 | 0.7315 | 0.4866 | -0.0098 | 0.1722 | -0.1004 | -4.1282 | 3.3013 | 0.17408 | -4.1730 | 3.2522 | 0.28 | 0.32 |
| S2 | 0.8669 | 0.7183 | 0.0016 | 0.5741 | 5.3817 | 3.7410 | -0.1871 | 5.8856 | 3.8104 | -0.4112 | 0.55 | 0.83 |
| S3 | 0.8179 | 0.6687 | 0.0003 | 0.4871 | 4.6297 | 1.0043 | -0.1194 | 3.9683 | 0.9793 | -0.1411 | 0.66 | 0.93 |
| S4 | 0.6690 | 0.6553 | 0.0026 | 0.1241 | 3.5005 | 4.8091 | 0.7625 | 5.5483 | 6.3044 | -0.2099 | 2.71 | 3.39 |
| S5 | 0.8271 | 0.6151 | -0.0006 | 0.5537 | 5.6641 | 5.7582 | -0.2859 | 5.8251 | 6.0081 | -0.3272 | 0.30 | 0.40 |
| S6 | 0.7346 | 0.5884 | 0.0065 | 0.3309 | 3.3694 | -1.9550 | 0.0880 | 0.8709 | -2.1188 | 0.3662 | 2.51 | 3.14 |
| S7 | 0.6741 | 0.5022 | 0.0006 | 0.4078 | 3.9918 | -1.8714 | -0.2232 | 4.1865 | -2.3397 | -0.2640 | 0.50 | 0.56 |
| S8 | 0.6741 | 0.5022 | 0.0006 | 0.4078 | 3.9918 | -1.8714 | -0.2232 | 4.1865 | -2.3397 | -0.2640 | 0.50 | 0.56 |
| S9 | 0.6252 | 0.4856 | -0.0042 | 0.3631 | 1.0778 | 0.8006 | 0.2322 | 1.9097 | 0.4323 | 0.3107 | 0.91 | 0.95 |
| S10 | 0.5944 | 0.5098 | -0.0010 | 0.2346 | 4.0912 | -4.2746 | -0.0291 | 4.5071 | -4.4989 | -0.6514 | 0.78 | 0.81 |

**Table S8:** Computed hole and electron overlap (S), distance between centroids of hole and electron (D, Å) and dipole moment (μ) for singlet states of CCAPI.

| State | Hole integral | Electron integral | Integral of transition density | Integral overlap of $h^{+}$- $e^{-}$ (S) | Centroid of hole (Å) | | | Centroid of electron (Å) | | | D (Å) | μ (a.u) |
|---|---|---|---|---|---|---|---|---|---|---|---|---|
| | | | | | x | y | z | x | y | z | | |
| T1 | 0.7421 | 0.6001 | -0.0002 | 0.5101 | 5.8538 | 6.2305 | -0.2957 | 5.8395 | 6.1302 | -0.3369 | 0.11 | 0.14 |
| T2 | 0.7055 | 0.5630 | -0.0003 | 0.4740 | 5.7616 | -5.1761 | -1.0074 | 5.7379 | -5.1737 | -0.9329 | 0.08 | 0.09 |
| T3 | 0.4008 | 0.3127 | -0.0051 | 0.2151 | 1.5584 | 0.6390 | 0.8530 | 1.0215 | 0.5173 | 0.8923 | 0.55 | 0.37 |
| T4 | 0.3356 | 0.2333 | -0.0065 | 0.1829 | -1.7035 | -0.9262 | 0.6226 | -2.1787 | -1.1937 | 0.5599 | 0.55 | 0.29 |
| T5 | 0.3180 | 0.2361 | 0.0044 | 0.1715 | -4.7701 | 0.5602 | 0.5347 | -3.5841 | 0.2954 | 0.7936 | 1.24 | 0.65 |
| T6 | 0.5577 | 0.3901 | -0.0030 | 0.3226 | 4.6288 | -4.4003 | -0.8903 | 5.0215 | -4.7702 | -0.8707 | 0.54 | 0.48 |
| T7 | 0.2912 | 0.1933 | -0.0072 | 0.1507 | -8.0548 | 1.3346 | -0.6761 | -9.5778 | 1.6501 | -1.1251 | 1.62 | 0.74 |
| T8 | 0.1888 | 0.1292 | -0.0082 | 0.0797 | -3.6189 | 0.4868 | 0.3305 | -3.4437 | 0.2588 | 0.9597 | 0.69 | 0.21 |
| T9 | 0.5766 | 0.3617 | 0.0006 | 0.3249 | -8.9966 | -1.3163 | -1.1419 | -10.0492 | -1.0531 | -1.3935 | 1.11 | 0.99 |
| T10 | 0.7935 | 0.5462 | -0.0009 | 0.5051 | 5.8344 | 6.3749 | -0.3711 | 5.7555 | 6.2335 | -0.3786 | 0.16 | 0.20 |

**Table S9:** Computed RMSD of electron and hole, H index and t index for singlet states of CAPI.

| State | RMSD (Electron) | | | | RMSD (Hole) | | | | H index | | | | t index | | | |
|---|---|---|---|---|---|---|---|---|---|---|---|---|---|---|---|---|
| | **x** | **y** | **z** | **total** | **x** | **y** | **z** | **total** | **x** | **y** | **z** | **Total** | **x** | **y** | **z** | **Total** |
| S1 | 1.634 | 1.699 | 1.314 | 2.699 | 1.526 | 1.709 | 0.967 | 2.487 | 1.580 | 1.704 | 1.140 | 2.588 | -1.269 | -1.419 | -1.058 | 2.178 |
| S2 | 2.293 | 5.232 | 0.714 | 5.757 | 2.756 | 4.755 | 0.740 | 5.545 | 2.524 | 4.993 | 0.727 | 5.642 | -1.917 | -4.919 | -0.664 | 5.321 |
| S3 | 4.052 | 5.696 | 0.757 | 7.031 | 3.330 | 5.307 | 0.782 | 6.314 | 3.691 | 5.501 | 0.770 | 6.669 | -3.565 | -5.168 | -0.672 | 6.314 |
| S4 | 2.523 | 3.339 | 0.706 | 4.244 | 3.494 | 4.129 | 0.741 | 5.460 | 3.009 | 3.734 | 0.723 | 4.850 | -2.194 | -2.672 | -0.676 | 3.523 |
| S5 | 4.634 | 3.770 | 0.802 | 6.028 | 3.762 | 4.094 | 0.810 | 5.619 | 4.198 | 3.932 | 0.806 | 5.808 | -2.166 | -3.723 | -0.715 | 4.366 |
| S6 | 3.600 | 4.245 | 0.772 | 5.619 | 2.744 | 4.155 | 0.769 | 5.038 | 3.172 | 4.200 | 0.770 | 5.319 | -2.669 | -3.912 | -0.764 | 4.797 |
| S7 | 3.394 | 4.481 | 0.791 | 5.676 | 3.475 | 4.587 | 0.842 | 5.816 | 3.435 | 4.534 | 0.816 | 5.746 | -3.291 | -4.373 | -0.771 | 5.527 |
| S8 | 1.926 | 2.668 | 0.687 | 3.361 | 1.801 | 2.459 | 0.467 | 3.084 | 1.863 | 2.563 | 0.577 | 3.221 | 0.468 | -1.111 | -0.378 | 1.263 |
| S9 | 4.596 | 3.320 | 0.804 | 5.727 | 5.144 | 3.570 | 0.854 | 6.319 | 4.870 | 3.445 | 0.829 | 6.023 | -3.931 | -3.135 | -0.548 | 5.058 |
| S10 | 4.717 | 3.140 | 0.858 | 5.731 | 5.051 | 3.287 | 0.858 | 6.087 | 4.884 | 3.214 | 0.858 | 5.909 | -4.319 | -2.844 | -0.858 | 5.242 |
| S11 | 3.459 | 1.944 | 0.865 | 4.061 | 2.710 | 2.265 | 0.749 | 3.611 | 3.085 | 2.104 | 0.807 | 3.820 | -1.692 | -1.954 | -0.761 | 2.694 |
| S12 | 2.392 | 2.032 | 1.395 | 3.434 | 3.782 | 2.409 | 1.042 | 4.603 | 3.087 | 2.220 | 1.218 | 3.993 | -2.833 | -0.660 | -1.195 | 3.145 |
| S13 | 4.646 | 2.168 | 0.833 | 5.194 | 5.377 | 2.088 | 0.874 | 5.834 | 5.012 | 2.128 | 0.854 | 5.511 | -3.534 | -2.040 | -0.812 | 4.161 |

**Table S10:** Computed RMSD of electron and hole, H index and t index for triplet states of CAPI.

| State | RMSD (Electron) | | | | RMSD (Hole) | | | | H index | | | | t index | | | |
|---|---|---|---|---|---|---|---|---|---|---|---|---|---|---|---|---|
| | x | y | z | total | x | y | z | total | x | y | z | Total | x | y | z | Total |
| T1 | 2.159 | 2.947 | 0.696 | 3.719 | 2.459 | 2.799 | 0.723 | 3.795 | 2.309 | 2.873 | 0.709 | 3.753 | -2.121 | -2.859 | -0.699 | 3.628 |
| T2 | 2.476 | 3.020 | 0.746 | 3.976 | 2.223 | 2.707 | 0.749 | 3.582 | 2.349 | 2.864 | 0.747 | 3.779 | -2.093 | -2.519 | -0.708 | 3.351 |
| T3 | 4.058 | 2.406 | 0.818 | 4.788 | 4.273 | 2.532 | 0.846 | 5.038 | 4.165 | 2.469 | 0.832 | 4.913 | -3.389 | -2.415 | -0.810 | 4.240 |
| T4 | 4.044 | 2.856 | 0.938 | 5.039 | 4.151 | 3.038 | 0.947 | 5.230 | 4.097 | 2.947 | 0.943 | 5.134 | -3.462 | -2.578 | -0.815 | 4.393 |
| T5 | 4.309 | 3.164 | 0.997 | 5.438 | 3.777 | 3.146 | 0.973 | 5.011 | 4.043 | 3.155 | 0.985 | 5.222 | -3.676 | -2.770 | -0.842 | 4.679 |
| T6 | 2.627 | 3.355 | 0.788 | 4.334 | 2.501 | 3.035 | 0.780 | 4.009 | 2.564 | 3.195 | 0.784 | 4.171 | -2.506 | -2.896 | -0.754 | 3.903 |
| T7 | 4.515 | 2.944 | 1.101 | 5.502 | 5.293 | 3.148 | 1.054 | 6.247 | 4.904 | 3.046 | 1.077 | 5.873 | -4.142 | -2.727 | -1.050 | 5.069 |
| T8 | 3.628 | 2.610 | 1.135 | 4.611 | 5.159 | 2.972 | 1.083 | 6.052 | 4.393 | 2.791 | 1.109 | 5.322 | -3.053 | -2.735 | -1.030 | 4.226 |
| T9 | 1.783 | 1.890 | 1.373 | 2.938 | 1.495 | 1.659 | 0.956 | 2.430 | 1.639 | 1.774 | 1.165 | 2.682 | -1.308 | -1.620 | -1.155 | 2.381 |
| T10 | 2.475 | 2.669 | 0.707 | 3.708 | 3.287 | 2.375 | 0.755 | 4.125 | 2.881 | 2.522 | 0.731 | 3.898 | -2.613 | -2.424 | -0.727 | 3.638 |
| T11 | 2.979 | 2.016 | 0.966 | 3.724 | 4.095 | 2.145 | 1.028 | 4.736 | 3.537 | 2.081 | 0.997 | 4.223 | -2.732 | -1.884 | -0.964 | 3.456 |
| T12 | 2.019 | 1.700 | 1.385 | 2.981 | 1.951 | 2.047 | 1.228 | 3.083 | 1.985 | 1.874 | 1.307 | 3.026 | -1.936 | -1.354 | -1.302 | 2.697 |
| T13 | 3.076 | 2.125 | 0.799 | 3.823 | 3.632 | 2.092 | 0.870 | 4.281 | 3.354 | 2.109 | 0.834 | 4.049 | -3.159 | -1.795 | -0.813 | 3.724 |

**Table S11:** Computed RMSD of electron and hole, H index and t index for singlet states of CCAPI

| State | RMSD (Electron) | | | | RMSD (Hole) | | | | H index | | | | t index | | | |
|---|---|---|---|---|---|---|---|---|---|---|---|---|---|---|---|---|
| | x | y | z | total | x | y | Z | total | x | y | z | Total | x | Y | Z | Total |
| S1 | 1.660 | 1.924 | 1.473 | 2.938 | 1.435 | 1.677 | 1.132 | 2.481 | 1.548 | 1.801 | 1.303 | 2.708 | -1.273 | -1.756 | -1.253 | 2.505 |
| S2 | 2.171 | 5.423 | 1.111 | 5.946 | 2.464 | 4.943 | 1.187 | 5.649 | 2.318 | 5.183 | 1.149 | 5.792 | -1.814 | -5.113 | -0.925 | 5.504 |
| S3 | 4.442 | 5.673 | 1.375 | 7.335 | 3.297 | 5.437 | 1.324 | 6.495 | 3.869 | 5.555 | 1.349 | 6.903 | -3.208 | -5.530 | -1.328 | 6.530 |
| S4 | 2.660 | 2.791 | 1.006 | 3.984 | 1.505 | 2.085 | 0.601 | 2.641 | 2.082 | 2.438 | 0.804 | 3.305 | -0.034 | -0.943 | 0.169 | 0.958 |
| S5 | 2.469 | 3.169 | 0.967 | 4.132 | 2.662 | 3.523 | 1.036 | 4.536 | 2.565 | 3.346 | 1.002 | 4.334 | -2.404 | -3.096 | -0.960 | 4.036 |
| S6 | 5.363 | 3.553 | 1.562 | 6.620 | 4.018 | 3.904 | 1.463 | 5.790 | 4.690 | 3.728 | 1.512 | 6.179 | -2.192 | -3.564 | -1.234 | 4.362 |
| S7 | 3.778 | 4.559 | 1.326 | 6.068 | 4.155 | 4.676 | 1.402 | 6.411 | 3.967 | 4.618 | 1.364 | 6.238 | -3.772 | -4.149 | -1.323 | 5.761 |
| S8 | 3.778 | 4.559 | 1.326 | 6.068 | 4.155 | 4.676 | 1.402 | 6.411 | 3.967 | 4.618 | 1.364 | 6.238 | -3.772 | -4.149 | -1.323 | 5.761 |
| S9 | 5.127 | 4.379 | 1.387 | 6.884 | 5.977 | 4.273 | 1.346 | 7.470 | 5.552 | 4.326 | 1.366 | 7.170 | -4.720 | -3.958 | -1.288 | 6.293 |
| S10 | 4.198 | 3.582 | 1.219 | 5.652 | 2.315 | 2.410 | 1.092 | 3.516 | 3.257 | 2.996 | 1.156 | 4.574 | -2.841 | -2.772 | -0.533 | 4.005 |

**Table S12:** Computed RMSD of electron and hole, H index and t index for triplet states of CCAPI.

| State | RMSD (Electron) | | | | RMSD (Hole) | | | | H index | | | | t index | | | |
|---|---|---|---|---|---|---|---|---|---|---|---|---|---|---|---|---|
| | x | y | z | total | x | y | z | total | x | y | z | Total | x | y | z | Total |
| T1 | 2.624 | 3.094 | 1.037 | 4.187 | 2.364 | 2.862 | 1.057 | 3.859 | 2.494 | 2.978 | 1.047 | 4.023 | -2.480 | -2.877 | -1.006 | 3.929 |
| T2 | 2.842 | 2.894 | 1.190 | 4.227 | 3.136 | 3.035 | 1.143 | 4.512 | 2.989 | 2.964 | 1.167 | 4.368 | -2.965 | -2.962 | -1.092 | 4.331 |
| T3 | 4.110 | 2.814 | 1.180 | 5.119 | 3.858 | 2.629 | 1.118 | 4.801 | 3.984 | 2.721 | 1.149 | 4.960 | -3.447 | -2.599 | -1.110 | 4.458 |
| T4 | 4.604 | 2.831 | 1.280 | 5.554 | 4.463 | 3.129 | 1.224 | 5.586 | 4.534 | 2.980 | 1.252 | 5.568 | -4.058 | -2.712 | -1.189 | 5.024 |
| T5 | 5.189 | 2.550 | 1.215 | 5.908 | 5.504 | 2.271 | 1.256 | 6.085 | 5.346 | 2.411 | 1.235 | 5.993 | -4.160 | -2.146 | -0.976 | 4.782 |
| T6 | 4.224 | 3.042 | 1.239 | 5.351 | 4.932 | 3.623 | 1.256 | 6.247 | 4.578 | 3.333 | 1.247 | 5.798 | -4.185 | -2.963 | -1.228 | 5.273 |
| T7 | 4.955 | 2.624 | 1.285 | 5.752 | 5.826 | 2.598 | 1.480 | 6.549 | 5.390 | 2.611 | 1.383 | 6.147 | -3.867 | -2.295 | -0.934 | 4.593 |
| T8 | 4.940 | 2.558 | 1.415 | 5.741 | 6.268 | 3.113 | 1.451 | 7.148 | 5.604 | 2.836 | 1.433 | 6.442 | -5.429 | -2.608 | -0.804 | 6.076 |
| T9 | 4.760 | 2.275 | 1.311 | 5.436 | 5.536 | 2.228 | 1.452 | 6.142 | 5.148 | 2.251 | 1.382 | 5.786 | -4.095 | -1.988 | -1.130 | 4.691 |
| T10 | 3.197 | 2.734 | 1.092 | 4.346 | 2.988 | 2.532 | 1.108 | 4.071 | 3.093 | 2.633 | 1.100 | 4.208 | -3.014 | -2.492 | -1.093 | 4.060 |

**Table S13:** Computed excitation energy (eV), excitation coefficient and Δr intex (Å) for singlet and triplet states of CAPI.

| State | Singlet | | | Triplet | | |
|---|---|---|---|---|---|---|
| | **Excitation energy** | **Excitation coefficient** | **Δrintex** | **Excitation energy** | **Excitation coefficient** | **Δrintex** |
| 1 | 2.5530 | 0.4603 | 0.4899 | 0.9256 | 0.4023 | 1.0539 |
| 2 | 2.6992 | 0.3748 | 2.9298 | 1.1544 | 0.3234 | 0.8482 |
| 3 | 2.8223 | 0.4176 | 4.0613 | 1.6697 | 0.3164 | 3.0507 |
| 4 | 3.1916 | 0.4330 | 1.5176 | 1.8088 | 0.3458 | 2.4732 |
| 5 | 3.3546 | 0.3736 | 4.1303 | 1.8418 | 0.3469 | 3.1039 |
| 6 | 3.4380 | 0.3970 | 1.8933 | 1.8777 | 0.3159 | 2.2818 |
| 7 | 3.5104 | 0.3869 | 1.3137 | 1.9730 | 0.1954 | 4.0896 |
| 8 | 3.6046 | 0.3718 | 2.5503 | 2.1282 | 0.2801 | 1.7987 |
| 9 | 3.7410 | 0.3763 | 3.1057 | 2.4177 | 0.1685 | 3.4267 |
| 10 | 3.7958 | 0.3337 | 3.9636 | 2.4845 | 0.1831 | 2.8224 |
| 11 | 3.8584 | 0.3409 | 6.7839 | 2.6116 | 0.2032 | 1.7256 |
| 12 | 3.9009 | 0.3430 | 6.4062 | 2.7331 | 0.3423 | 2.4644 |
| 13 | 3.9366 | 0.3748 | 7.5776 | 2.8288 | 0.2432 | 2.3739 |

**Table S14:** Computed excitation energy (eV), excitation coefficient and Δr intex (Å) for singlet and triplet states of CCAPI.

| State | Singlet | | | Triplet | | |
|---|---|---|---|---|---|---|
| | **Excitation energy** | **Excitation coefficient** | **Δrintex** | **Excitation energy** | **Excitation coefficient** | **Δrintex** |
| 1 | 1.5235 | 0.4722 | 4.8646 | 1.0082 | 0.9190 | 1.6722 |
| 2 | 1.6700 | 0.4318 | 3.2430 | 1.2123 | 0.8942 | 2.5104 |
| 3 | 1.9815 | 0.4255 | 3.2524 | 1.2260 | 0.6886 | 1.7461 |
| 4 | 2.0923 | 0.4295 | 2.0114 | 1.4861 | 0.8318 | 2.2515 |
| 5 | 3.0029 | 0.4384 | 3.3769 | 1.6877 | 0.6374 | 3.1975 |
| 6 | 3.1225 | 0.4249 | 3.6046 | 1.8333 | 0.5761 | 2.6002 |
| 7 | 3.2380 | 0.4444 | 3.4306 | 1.9661 | 0.4471 | 1.6610 |
| 8 | 3.3551 | 0.3970 | 1.1773 | 2.0910 | 0.6773 | 2.1668 |
| 9 | 3.4602 | 0.3677 | 3.9322 | 2.1579 | 0.6372 | 2.8291 |
| 10 | 3.4743 | 0.4065 | 3.2327 | 2.2018 | 0.5635 | 2.3571 |

**Table S15.** Transferred charges ($q_{CT}$), barycentres of electron density loss ($R_+$) /gain (R-), distance between two barycenters ($D_{CT}$), dipole moment of CT ($\mu_{CT}$), RMSD of +ve/-ve parts, CT indices (H & t) and overlap integral of C+/C- of CAPI and CCAPI.

| Blue emissive & Host materials | $q_{CT}$ $\lvert e^{-1} \rvert$ | $R_+$ (Å) | | | R- (Å) | | | $D_{CT}$ (Å) | $\mu_{CT}$ (D) | RMSD of +ve parts | RMSD of -ve parts | H / t indices (Å) | overlap integral of C+/ C- |
|---|---|---|---|---|---|---|---|---|---|---|---|---|---|
| | | x | y | z | x | y | z | | | | | | |
| **CAPI** | 23.347 -22.197 | 1.432 | 0.194 | -0.099 | 1.210 | 0.441 | -0.099 | 0.332 | 37.284 | 10.964 | 11.408 | 5.919/5.618 | 0.9980 |
| **CCAPI** | 23.594 -22.488 | 0.881 | -0.406 | -0.150 | 0.885 | -0.273 | -0.147 | 0.133 | 15.068 | 11.257 | 11.106 | 5.916/5.858 | 0.9962 |
